# Neural Regulation of Cardiac Arrhythmias: From the Brain–Heart Axis to Emerging Precision Therapies

**DOI:** 10.34133/research.1340

**Published:** 2026-07-01

**Authors:** Yudong Xia, Yu Han, Tao Yu, Li Ni, Dao Wen Wang

**Affiliations:** ^1^ Division of Cardiology, Department of Internal Medicine, Tongji Hospital, Tongji Medical College, Huazhong University of Science and Technology, Wuhan 430030, China.; ^2^ Hubei Key Laboratory of Genetics and Molecular Mechanisms of Cardiological Disorders, Wuhan 430030, China.; ^3^ Institute for Translational Medicine, The Affiliated Hospital of Qingdao University, Qingdao 266021, China.

## Abstract

Historically, cardiac arrhythmias have been interpreted as intrinsic ion channel disorders of the cardiomyocyte. However, the field is currently undergoing a fundamental paradigm shift, recognizing the brain–heart axis as the ultimate arbiter of electrical stability. Despite a wealth of preclinical evidence, the clinical translation of neuromodulatory interventions has reached a critical juncture, with many trials yielding conflicting or disappointing outcomes. This translational gap underscores a critical need to move beyond an oversimplified, efferent-centric view toward an integrated understanding of the neuro-cardiac hierarchy. This review redefines arrhythmogenesis as an emergent property of multi-level network instability, driven by 3 core mechanistic principles: spatial heterogeneity, temporal dynamics, and convergent remodeling. By synthesizing the anatomical architecture of the cardiac autonomic nervous system, integrating the often-overlooked afferent sensory limb and the intrinsic “little brain”, we illustrate how neural circuits translate systemic stressors into localized electrophysiological triggers. We examine the bidirectional neuroimmune crosstalk and neuropeptide dynamics that bridge inflammatory signaling with maladaptive remodeling. Furthermore, we explore how circadian rhythms impose temporal windows of vulnerability, transforming stable substrates into arrhythmogenic ones. Finally, we critically evaluate the therapeutic landscape, from established pharmacological blockade to the nascent frontiers of closed-loop bioelectronics and nanotechnology. By bridging molecular mechanisms with a hierarchical appraisal of clinical evidence, this review provides a roadmap for the next generation of precision neuromodulation designed to restore and sustain neuro-cardiac homeostasis.

## Introduction

Cardiac arrhythmias have traditionally been interpreted as disorders arising primarily from intrinsic ion channel dysfunction in cardiomyocytes [1]. However, accumulating evidence indicates that arrhythmogenesis is governed by complex multi-system interactions involving neural, endocrine, and immune regulatory networks along the brain–heart axis [1]. Accordingly, cardiac arrhythmias are increasingly recognized as multi-system disorders arising from a hierarchical interplay between intrinsic cardiac properties and neural regulatory circuits [2,3].

Cardiac electrophysiology is coordinated by an integrated neural network linking higher cortical centers with the extrinsic and intrinsic cardiac nervous systems (ECNS and ICNS) [4,5]. The ICNS, embedded within epicardial fat pads and organized into ganglionated plexi (GPs), acts as a local neural processing center that modulates autonomic inputs to the myocardium [6,7]. Together with neuroimmune signaling pathways, these neural circuits maintain the spatiotemporal stability of cardiac electrophysiology under physiological conditions [8]. The cardiac autonomic nervous system (CANS) preserves cardiovascular homeostasis by balancing sympathetic and parasympathetic influences on heart rate, conduction, and contractility [9].

In pathological states such as myocardial infarction (MI) and heart failure (HF), this regulatory system undergoes maladaptive remodeling characterized by regional denervation, sympathetic hyperinnervation, and neurohumoral activation [10]. These alterations disrupt electrical stability through heterogeneous autonomic signaling, abnormal Ca^2+^ handling, and structural remodeling, thereby increasing the susceptibility to arrhythmias [11–13]. Moreover, autonomic imbalance and neural dysregulation contribute to the circadian patterning of arrhythmic events, reflecting dynamic interactions between central neural control and cardiac electrophysiology [14].

These insights have prompted a paradigm shift in arrhythmia research, moving beyond the traditional ion channel-centric view toward an integrated perspective of neuro-cardiac regulation. Consequently, therapeutic strategies are increasingly targeting the neural and systemic drivers of arrhythmogenesis. Emerging neuromodulatory approaches, spanning from the pharmacological modulation of neurohumoral pathways to autonomic interventions such as renal denervation (RDN) and left cardiac sympathetic denervation (LCSD), as well as bioelectronic therapies like vagus nerve stimulation (VNS), are reshaping the therapeutic landscape of arrhythmia management [15–20]. In addition, behavioral interventions that modulate autonomic tone, including heart rate variability (HRV) biofeedback and mind–body practices, offer complementary top-down approaches to regulate the brain–heart axis [21,22].

Prior reviews have established the foundational framework of neuro-cardiac regulation and highlighted the clinical complexities of translating autonomic mechanisms into therapeutic interventions [1,13]. The present review advances the field in 3 distinct respects. First, we emphasize the hierarchical and integrative nature of neuro-cardiac regulation, reframing arrhythmogenesis as an emergent property of multi-level network instability rather than isolated pathway dysfunction. Second, we incorporate recent advances in neuroimmune crosstalk, circadian-dependent vulnerability, and co-transmitter signaling, including the role of neuropeptide Y (NPY) in sympathovagal decoupling. Third, we extend the therapeutic discussion beyond established pharmacological and surgical neuromodulation to encompass next-generation closed-loop bioelectronic systems, nanotechnology-enabled neural interfaces, and behavioral interventions while critically appraising the translational status and evidentiary support for each strategy.

To deliver on these aims, the review is organized around 3 mechanistic principles that collectively explain how neural dysregulation translates into arrhythmogenesis. The first principle is spatial heterogeneity: Regional differences in innervation density, lateralized specialization of right versus left autonomic inputs, and the patchy denervation and hyperinnervation that follow injury create spatial dispersion of refractoriness and conduction, establishing the substrate for reentry and triggered activity. The second principle is temporal dynamics: Autonomic outflow oscillates across multiple timescales, from beat-to-beat baroreflex adjustments to circadian rhythms; disruption of these patterns generates transient windows of vulnerability in which otherwise stable substrates become arrhythmogenic. The third principle is convergent remodeling: neural, humoral, and immune pathways converge upon common molecular effectors, driving ion channel remodeling, gap junction dysfunction, and fibrosis in a self-perpetuating cycle of electrical instability. By applying these principles, we reinterpret the anatomical organization of the CANS, the molecular transduction of neural signals, the maladaptive remodeling of disease, and the therapeutic opportunities afforded by neuromodulation. This integrated framework redefines arrhythmias not as isolated ion channel disorders but as emergent consequences of neuro-cardiac network instability, highlighting the need for mechanism-guided, individualized, mechanism-matched therapeutic strategies.

In the following sections, we first outline the anatomical and functional organization of the CANS, then examine the molecular mechanisms linking neurohumoral and neuroimmune signaling to electrophysiological remodeling, followed by a discussion of maladaptive neural remodeling in MI and HF. We conclude by surveying emerging neuromodulatory therapies, from pharmacological interventions to next-generation bioelectronic systems.

## Functional Hierarchy of the CANS

This section outlines the anatomical basis of the CANS and explains how regional differences in innervation density and lateralized function create the spatial heterogeneity that influences arrhythmia susceptibility. We first describe the extrinsic sympathetic and parasympathetic pathways, then focus on the ICNS as a local integration hub, and finally examine cardiac afferent neurons as the sensory limb that initiates reflexes modulating efferent autonomic tone and contributing to arrhythmogenesis when dysregulated.

**Fig. 1.**
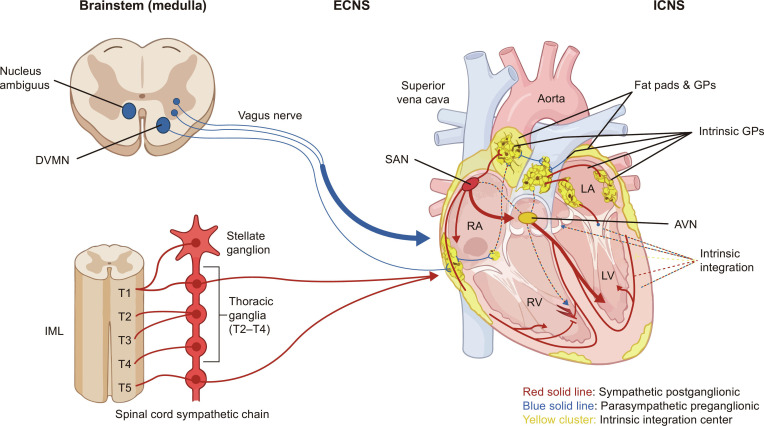
Anatomical organization and integrated regulatory network of the extrinsic cardiac nervous system (ECNS) and intrinsic cardiac nervous system (ICNS). The ECNS originates from central command centers. Parasympathetic preganglionic fibers (solid blue lines) arise from brainstem nuclei, specifically the nucleus ambiguus and the dorsal vagal motor nucleus (DVMN), traveling via the vagus nerve. Sympathetic preganglionic fibers arise from the spinal cord IML column (T1 to T5) and synapse in paravertebral ganglia, including the stellate ganglion and thoracic ganglia (T2 to T4). Sympathetic postganglionic fibers (solid red lines) project to the heart. Functionally, right-sided sympathetic fibers predominantly innervate the sinoatrial node (SAN) to control heart rate (chronotropic effect), whereas left-sided fibers exert a stronger influence on the atrioventricular node (AVN) to modulate conduction velocity (dromotropic effect). The ICNS consists of a complex network of neurons organized into intrinsic ganglionated plexi (GPs) (yellow clusters) located within epicardial fat pads. These GPs serve as local integration centers that receive and process extensive inputs from both extrinsic sympathetic and parasympathetic nerves before relaying integrated signals (dotted mixed-color lines) to specific cardiac targets, such as SAN, atrioventricular node (AVN), and ventricular myocardium. IML, intermediolateral column; RA, right atrium; RV, right ventricle; LA, left atrium; LV, left ventricle.

Cardiac function is orchestrated by a hierarchical autonomic network comprising integrated extrinsic and intrinsic components as schematically depicted in Fig. 1. This hierarchical architecture embodies the principle of spatial heterogeneity: Sympathetic and parasympathetic fibers exhibit distinct topographical distributions, with right- and left-sided inputs displaying functional lateralization that differentially influences nodal and ventricular electrophysiology. The following subsections detail how this anatomical and functional organization establishes the spatially nonuniform autonomic landscape upon which arrhythmogenic substrates are superimposed in disease.

By integrating antagonistic sympathetic and parasympathetic outflows, this network exerts exquisite control over chronotropic, dromotropic, and inotropic states. While the ECNS relays central and peripheral commands from the brain and thoracic ganglia, the ICNS, which is embedded within epicardial fat pads, functions as a localized micro-circuitry that integrates, filters, and modulates these inputs. Collectively, this hierarchical architecture ensures the precise spatiotemporal coordination of autonomic tone across discrete cardiac domains, enabling the heart to adapt to fluctuating physiological demands.

### Extrinsic sympathetic regulation: Structural and functional landscapes

Cardiac sympathetic efferents originate from paravertebral autonomic ganglia situated along the cervical and upper thoracic spinal cord. This neural chain encompasses the superior cervical ganglion, the stellate (cervicothoracic) ganglion, and the thoracic sympathetic ganglia, which extend caudally to the T7 vertebral level. Postganglionic sympathetic cell bodies are predominantly localized within these ganglia, giving rise to axons that coalesce into the superior, middle, and inferior cardiac nerves. These nerves subsequently project to the cardiac plexi, where they provide robust and comprehensive sympathetic innervation to the myocardium and conduction system [4]. The cardiac sympathetic efferent limb originates from preganglionic neurons residing within the intermediolateral (IML) cell column of the spinal cord at the T1**–**T4 levels. Their axons project to synapse with postganglionic neurons localized within the paravertebral ganglia of the lower cervical and upper thoracic regions. Characteristically, the inferior cervical and first thoracic (T1) ganglia coalesce to form the bilateral stellate ganglia. Morphological variations occur where the second thoracic (T2) ganglion also fuses into this complex, resulting in a trilobed configuration. Supplemental postganglionic sympathetic innervation is derived from paravertebral ganglia corresponding to the T2**–**T4 spinal segments, further contributing to the dense neural meshwork of the cardiac plexi [5].

Sympathetic terminals directly innervate cardiomyocytes and establish synaptic connections with neurons within the intrinsic cardiac ganglia (ICG). This neuro-anatomical configuration facilitates local signal integration and amplification within the heart’s own neural network. At the supraventricular level, right- and left-sided sympathetic efferents exhibit distinct yet overlapping topographical distributions. Convergent evidence from experimental models and clinical studies has firmly established that right-sided fibers exert a predominant chronotropic influence on the sinoatrial node (SAN), whereas left-sided nerves have a more pronounced dromotropic effect on the atrioventricular node (AVN). Collectively, these bilateral inputs provide coordinated regulation of atrial electrophysiology and ensure adaptive responses to changing physiological demands [9].

The pivotal role of right-sided sympathetic innervation in orchestrating cardiac chronotropy has been robustly established across both experimental models and clinical studies. Furthermore, recent advancements in precision neuro-engineering, particularly optogenetic modulation and viral vector-mediated circuit tracing in rodent and porcine models, have substantiated these findings with high spatiotemporal resolution. These high-fidelity preclinical tools have allowed for the selective manipulation of specific neural populations, confirming that right sympathetic efferents serve as the primary drivers of HRV and SAN excitability [23]. While direct optogenetic interrogation in humans is not currently feasible, the functional lateralization observed in these models aligns with clinical observations from stellate ganglion blockade and surgical denervation procedures. Specifically, ultrasound-guided bilateral stellate ganglion blockade has been shown to substantially reduce ventricular arrhythmia burden in patients with refractory electrical storm, whereas LCSD significantly decreases the incidence of aborted cardiac arrest and syncope in high-risk patients with long QT syndrome, with minimal effect on resting heart rate [24]. These clinical findings corroborate the preclinical evidence that right-sided sympathetic fibers predominantly govern chronotropy, while left-sided input exerts greater influence over ventricular repolarization and arrhythmic susceptibility.

This functional lateralization is even more pronounced within the ventricular myocardium. Traditionally, the anterior ventricular wall is considered to be predominantly innervated by right-sided sympathetic fibers, whereas the posterior wall remains under the primary governance of left-sided nerves [25]. Nevertheless, experimental evidence from canine and porcine models reveals substantial topographical overlap between right- and left-sided sympathetic distributions across the ventricles [26]. The extent to which this overlapping innervation pattern translates to human ventricular electrophysiology remains to be fully characterized. However, clinical cardiac sympathetic imaging studies using ^123^I-metaiodobenzylguanidine (^123^I-MIBG) single-photon emission computed tomography (SPECT) or ^11^C-meta-hydroxyephedrine (^11^C-HED) positron emission tomography (PET) have consistently demonstrated that regional heterogeneity of sympathetic denervation independently predicts the risk of ventricular arrhythmias and sudden cardiac arrest in patients with ischemic cardiomyopathy [27], suggesting that the spatial organization of ventricular sympathetic innervation carries functionally significant implications for arrhythmogenesis in humans. Microanatomically, sympathetic efferents course in parallel with the coronary arteries and are primarily sequestered within the subepicardial layer. Studies across multiple species consistently delineate 2 prominent innervation gradients: a longitudinal base-to-apex decline in nerve density and a transmural decrease from the subepicardium toward the subendocardium [28]. Nevertheless, significant regional heterogeneities exist. For instance, the canine right ventricular outflow tract (RVOT) exhibits dense sympathetic innervation across both subepicardial and subendocardial planes [29]. Furthermore, despite their subendocardial localization, the papillary muscles are richly innervated, facilitating robust inotropic responses during sympathetic arousal. Notably, the posterior papillary muscle is under the primary governance of left-sided sympathetic nerves, whereas the anterior papillary muscle receives dual bilateral inputs [30]. The specialized conduction system, including the SAN, AVN, and bundle of His, possesses a significantly higher innervation density than the working myocardium, receiving the high-fidelity dual-autonomic inputs required for precise regulatory control [30].

### Parasympathetic pathways: Integrative conduction and vagal control

Cardiac parasympathetic innervation mainly arises from the nucleus ambiguus in the medulla oblongata. Its preganglionic fibers travel almost entirely within the vagus nerve, which splits into superior, middle, and inferior cardiac branches. Most of these vagal fibers converge at a distinct fat pad, commonly known as the third fat pad, which is situated between the superior vena cava and the aorta. This site acts as a key convergence point, organizing parasympathetic input before it reaches and regulates the SAN and AVN [31]. The cell bodies of parasympathetic preganglionic neurons are mainly located in the dorsal vagal motor nucleus (DVMN) and the nucleus ambiguous. Their axons travel along the vagus nerve and terminate within the intrinsic cardiac GPs embedded in the epicardial fat pads, where they synapse with postganglionic cholinergic neurons. Notably, these postganglionic neurons can receive convergent preganglionic input from both the left and right vagal trunks [1].

Regarding the topographical bias of central parasympathetic output, evidence suggests that preganglionic neurons originating from the nucleus ambiguus preferentially modulate nodal structures, whereas those in DVMN exhibit a greater propensity toward ventricular regulation. However, this functional segregation is neither absolute nor exclusive; contemporary optogenetic studies corroborate a “preferential rather than selective” targeting paradigm [1]. Given the bilateral projection of preganglionic fibers across multiple GPs, the extensive inter-ganglionic connectivity, and the divergent terminal projections of postganglionic cholinergic neurons, cardiac parasympathetic regulation is best characterized by an integrative and divergent architecture rather than a region-specific, convergent control model [26,32]. This decentralized processing network ensures robust and flexible autonomic modulation of the heart across various physiological states.

### The ICNS: Local circuitry and integrative hubs

Complementing the extrinsic autonomic pathways, the heart is regulated by an intricately organized ICNS. Pioneering mapping of human cardiac innervation by Armour et al. [6] delineated a widespread distribution of ICG dispersed across the epicardial surfaces. Each ganglion typically harbors 200 to 1,000 neurons that serve as sophisticated synaptic relays for sympathetic and parasympathetic fibers entering the pericardial space. Most of these ganglia are clustered into GPs, which are sequestered within epicardial fat pads and interconnected by a dense network of axons [6,9]. These GPs function as local integrative hubs, orchestrating the complex cross-talk between extrinsic neural commands and intrinsic cardiac demands [7]. Consequently, elucidating the functional architecture of this cardiac neural network is imperative for deconstructing the mechanisms of arrhythmogenesis and paving the way for next-generation, pathology-specific neuromodulatory interventions.

### Cardiac afferent neurons: Sensory signaling and reflex arrhythmogenesis

While the preceding discussion has focused on efferent autonomic outflow, cardiac function is also profoundly influenced by afferent signaling from the heart to the central nervous system (CNS). The autonomic nervous system intricately regulates cardiac excitability and contractile function, with cardiac afferents providing beat-to-beat sensory information of cardiac muscle activity to the neuroaxis, while additional information is conveyed by extracardiac circulatory receptors [33]. These afferent fibers represent a critical sensory limb of the neuro-cardiac axis, initiating reflexes that dynamically modulate efferent autonomic tone.

Cardiac afferents are broadly classified into 2 populations based on their anatomical trajectory. Spinal (sympathetic) afferents have their cell bodies in the dorsal root ganglia (T1–T6) and project centrally to the upper thoracic and lower cervical spinal cord, whereas vagal (parasympathetic) afferents ascend to the left and right inferior vagal (nodose) ganglia and project to the nucleus tractus solitarius (NTS) in the brainstem [26,34]. Fibers from both sympathetic and parasympathetic branches mix to form the cardiac plexi, which projects a fine network over the heart below the pericardium and endocardium [26]. This anatomical organization underlies the cardiac–cardiac reflexes that provide constant feedback about cardiac function: Neural impulses traveling in vagal afferents elicit sympathoinhibition and hypotension, whereas impulses traveling in cardiac sympathetic afferents and spinal pathways elicit sympathoexcitation and hypertension [26].

Under pathological conditions, aberrant afferent signaling contributes directly to arrhythmogenesis. The processing of afferent information at several levels of the cardiac neuraxis, including the ICNS, extracardiac–intrathoracic ganglia, spinal cord, brainstem, and higher centers, provides an elegant mechanism for interacting feedback loops that maintain physiological stability in health [33]. However, cardiac disease results in adaptations of afferent input to various levels of the neuraxis, and such adaptations result in changes to integrated neural function within central and peripheral aspects of the cardiac nervous system [33]. Chronic sympathoexcitation is implicated in ventricular arrhythmogenesis following MI, and the cardiac sympathetic afferent reflex, partly mediated by fibers expressing the transient receptor potential cation subfamily V member 1 (TRPV1) channel, contributes to the initiation and persistence of adrenergic activation [35]. Afferent sensory transduction of the pathologically stressed heart results in a reflex-driven adrenergic efferent postganglionic neuronal output to the heart that is inherently proarrhythmic [33]. Furthermore, many intrinsic cardiac afferent neurons transduce the local mechanical and chemical milieu of the heart, and excessive activation of select elements within the ICNS can result in the genesis of atrial or ventricular arrhythmias [34]. Cardiac injury or functional abnormalities may also damage afferent neural pathways or alter the information transmitted along them, thereby creating conditions that lead to lethal arrhythmias [26].

In summary, cardiac afferent neurons constitute an essential sensory limb of the neuro-cardiac axis, participating in cardiac–cardiac reflexes that modulate sympathovagal balance. A comprehensive understanding of arrhythmia mechanisms therefore requires consideration of both afferent and efferent pathways, as well as the central integration of these signals within the cardiac neuronal hierarchy.

## Molecular Transduction and Neurohumoral Integration

This section applies the principles of temporal dynamics and convergent remodeling to the molecular effectors of neuro-cardiac signaling. It examines how neurotransmitters and circulating hormones act over distinct timescales to modulate cardiomyocyte excitability and how diverse upstream signals converge upon common Ca^2+^ handling and electrophysiological substrates. The following subsections detail these mechanisms, beginning with adrenergic and muscarinic signaling and progressing to the integrative roles of co-transmitters and systemic neurohumoral axes.

**Fig. 2.**
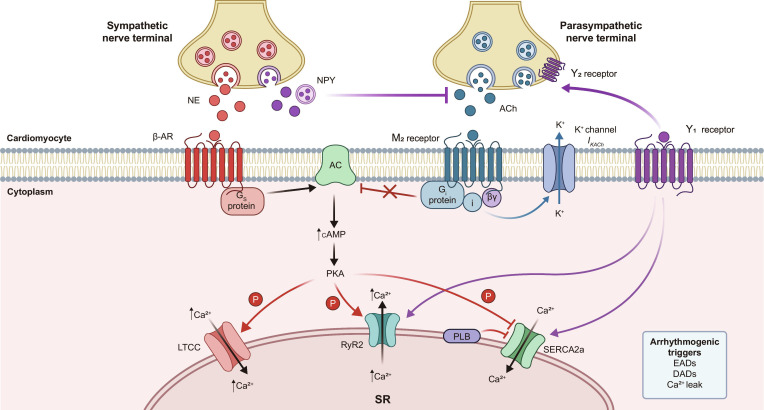
Integrated molecular pathways of neuro-cardiac signaling and cellular arrhythmogenic triggers. (Top) Presynaptic regulation: The sympathetic nerve terminal releases norepinephrine (NE) and the co-transmitter neuropeptide Y (NPY) upon stimulation, and the parasympathetic terminal releases acetylcholine (ACh). NPY acts presynaptically on Y_2_ receptors to inhibit ACh release, facilitating sympathetic-vagal decoupling. (Middle) Membrane receptors and signaling cascades: NE binds to β-adrenergic receptors (β-AR), activating the G_s_ protein–adenylyl cyclase (AC)–cyclic adenosine monophosphate (cAMP)–protein kinase A (PKA) cascade (red pathway). ACh binds to M_2_ muscarinic receptor, activating G_i_ protein that inhibits AC and activating ACh-activated K^+^ current (*I*_KACh_) (blue pathway). NPY binds to postsynaptic Y_1_ receptor (purple pathway). (Bottom) Ca^2+^ handling and arrhythmia triggers: PKA activation leads to phosphorylation (P) of key Ca^2+^-handling proteins: L-type Ca^2+^ channel (LTCC) (increasing Ca^2+^ influx), ryanodine receptor 2 (RyR2) [increasing sarcoplasmic reticulum (SR) Ca^2+^ release], and phospholamban (PLB) [relieving inhibition of sarco/endoplasmic reticulum Ca^2+^-ATPase 2a (SERCA2a), promoting Ca^2+^ reuptake]. Postsynaptic NPY signaling also modulates RyR2 and SERCA2a function. Under pathological conditions or excessive sympathetic drive, these alterations, particularly increased SR Ca^2+^ load and RyR2 leakiness mediated by PKA and NPY, can lead to diastolic Ca^2+^ leak, generating early afterdepolarizations (EADs) and delayed afterdepolarizations (DADs), which serve as critical triggers for arrhythmias.

Arrhythmogenic susceptibility is governed by a complex neuro-endocrine-immune network that translates systemic stressors into localized electrical instability. This regulatory hierarchy converges upon discrete molecular effectors, where autonomic neurotransmitters, neuropeptides, and circulating hormones modulate cardiomyocyte signaling cascades across diverse spatiotemporal scales. Furthermore, a recently proposed paradigm identifies intrinsic endogenous transmitter systems (ETSs), comprising glutamatergic, cholinergic, and GABAergic (γ-aminobutyric acid-ergic) networks, as a local bioelectric regulatory layer [36]. At the core of this integrated regulation is the synergistic interplay between the sympatho-adrenomedullary (SAM) axis and the renin–angiotensin–aldosterone system (RAAS), which orchestrates acute electrophysiological triggers and chronic structural remodeling [1]. By deconstructing the “double-edged sword” of adrenergic signaling, muscarinic antagonism, and NPY-mediated modulation, we can elucidate the mechanisms driving focal and re-entrant arrhythmias, specifically aberrant Ca^2+^ handling and repolarization dispersion [32,37]. Ultimately, this section explores how the integration of these multi-level signals precipitates a transition from physiological adaptation to a pathologically pro-arrhythmic state. The integrated molecular crosstalk and intracellular signaling cascades underlying these interactions are illustrated in Fig. 2.

### Adrenergic signaling: Drivers of contractility and arrhythmic triggers

Cardiomyocytes express both α-adrenergic receptors (α-ARs) and β-AR, the latter comprising the vast majority (~90%) of the myocardial adrenergic pool. In the healthy heart, the β_1_-AR to β_2_-AR ratio is maintained at approximately 4:1 (established in human myocardial samples). However, chronic cardiac stress and HF precipitate a profound remodeling of this landscape, notably through the selective down-regulation and desensitization of β_1_-AR [23,38] (primarily from preclinical models and human HF tissue studies). From the standpoint of neuro-endocrine integration, this receptor redistribution dictates that uniform catecholaminergic stimulation elicits fundamentally divergent electrophysiological responses in the diseased versus the healthy myocardium, potentially exacerbating arrhythmic vulnerability through altered intracellular signaling.

Stimulation of β₁-AR activates the canonical G_s_–adenylyl cyclase (AC)–cyclic adenosine monophosphate (cAMP)–protein kinase A (PKA) signaling axis, which orchestrates the classic sympathetic suite of positive chronotropic, inotropic, and dromotropic responses. Within the SAN, cAMP directly modulates hyperpolarization-activated cyclic nucleotide-gated (HCN) channels, enhancing the funny current (*I*_f_) and accelerating diastolic depolarization [39]. *I*_f_ is a mixed sodium–potassium inward current that activates during the slow diastolic depolarization phase in pacemaker cells, progressively driving the membrane potential toward the threshold that initiates the next heartbeat. Concurrently, PKA phosphorylates a strategic cluster of Ca^2+^-handling proteins, including LTCC, RyR2, and PLB. PKA-mediated phosphorylation of PLB disinhibits SERCA2a, acting in concert with augmented L-type Ca^2+^ current (*I*_CaL_) and enhanced SR Ca^2+^ loading to intensify myocardial contractility [40]. This “coupled-clock” mechanism is further refined by an intrinsic glutamatergic system, where endogenous glutamate serves as an igniter for local Ca^2+^ releases (LCRs) in SAN pacemaker cells. This signaling process promotes the oxidation of Ca^2+^-handling proteins, thereby driving the LCR activity essential for spontaneous firing [41].

Under pathological conditions, however, this augmented Ca^2+^ cycling can transition into a potent pro-arrhythmic substrate. Elevated SR Ca^2+^ sequestering, coupled with RyR2 hyperphosphorylation, precipitates spontaneous diastolic Ca^2+^ leak, a mechanism extensively characterized in isolated cardiomyocyte preparations and transgenic murine models. Evidence from human ventricular tissue obtained from patients with HF corroborates increased RyR2 phosphorylation and diastolic Ca^2+^ spark frequency, supporting the translational relevance of these preclinical findings [42]. The subsequent electrogenic extrusion of Ca^2+^ via the Na^+^/Ca^2+^ exchanger 1 (NCX1) generates a net inward depolarizing current (*I*_NCX_). In SAN myocytes, this mechanism accelerates the “Ca^2+^ clock” (part of the coupled-clock system), potentially driving inappropriate sinus tachycardia. In the working myocardium, these stochastic Ca^2+^ releases trigger delayed afterdepolarizations (DADs) or early afterdepolarizations (EADs), serving as a critical nidus for focal arrhythmias and premature beats. Furthermore, spatiotemporal heterogeneities in sympathetic innervation or neurotransmitter release exacerbate regional variances in β_1_-AR activation, profoundly amplifying nonuniformity in Ca^2+^ handling and repolarization dispersion [3].

Functionally, the positive dromotropic effects within the SAN and AVN are primarily mediated by augmented *I*_CaL_ density and kinetics. Beyond the nodes, accelerated intraventricular conduction is driven by PKA-dependent phosphorylation of voltage-gated Na^+^ channel (Na_v1.5_) and the acute and chronic modulation of connexin 43 (Cx43) proteostasis and gap junctional coupling [43]. To facilitate rate-dependent adaptation, the action potential duration (APD) shortens, largely through PKA-mediated enhancement of the slow delayed rectifier K^+^ current (*I*_Ks_) in large mammals [43]. Crucially, the impact of β-AR activation on arrhythmic stability is fundamentally contingent upon the spatiotemporal architecture of its activation. While acute, uniform sympathetic stimulation can suppress Ca^2+^/APD alternans and optimize electrical propagation, the transition to a pro-arrhythmic state is often dictated by the underlying substrate. In the context of preexisting Ca^2+^-handling instability or pronounced neural heterogeneities, these same signaling pathways exacerbate electrical nonuniformity and repolarization dispersion, thereby substantially heightening arrhythmic vulnerability [3].

### Muscarinic regulation: Counter-regulatory signaling and myocardial protection

While adrenergic signaling drives excitatory responses, the parasympathetic limb provides counter-regulation primarily through muscarinic acetylcholine receptors (mAChRs). mAChR are G protein-coupled receptors, with the M_2_ subtype serving as the predominant cardiac isoform, primarily signaling through G_i_ proteins. ACh released from parasympathetic terminals activates M_2_ receptors, which antagonize the β-AR–G_s_ axis by inhibiting AC and suppressing cAMP production. This signaling cascade elicits negative chronotropic, dromotropic, inotropic, and lusitropic responses. Furthermore, M_2_ activation potentiates *I*_KACh_ via the Gβγ subunit, inducing membrane hyperpolarization in nodal myocytes. Consequently, the synergism between *I*_KACh_ activation, *I*_f_ suppression, and “Ca^2+^ clock” deceleration drives the bradycardic response to parasympathetic stimulation [44]. In the atrial myocardium, these mechanisms concurrently abbreviate the APD, which may facilitate the genesis of atrial arrhythmias, such as atrial fibrillation (AF), under specific pathological conditions [45].

Beyond the canonical M_2_ isoform, cardiomyocytes express M_3_ muscarinic receptors, which contribute to a more nuanced regulatory landscape. Emerging evidence suggests that M_2_ and M_3_ receptors function synergistically to optimize ventricular Ca^2+^ homeostasis through distinct yet coordinated molecular pathways. Specifically, M_2_–G_i_ signaling activates protein kinase G (PKG) to modulate RyR2 at the Ser^2808^ site, while M_3_ activation facilitates dephosphorylation of the RyR2-Ser^2818^ site by suppressing reactive oxygen species (ROS)-dependent activation of Ca^2+^/calmodulin-dependent protein kinase II (CaMKII) [46]. This integrated signaling enhances systolic Ca^2+^ release efficiency without exacerbating diastolic SR Ca^2+^ leak. These findings underscore that parasympathetic regulation transcends mere passive antagonism of sympathetic activity; rather, it exerts active, independent, and protective control over myocardial electrophysiology and contractility.

### NPY and co-transmitters: Mediators of autonomic decoupling

NPY, a highly conserved 36-amino acid polypeptide, was initially isolated from the porcine brain and subsequently identified as a ubiquitous constituent of the central and peripheral nervous systems. Within the peripheral autonomic nervous system, NPY is localized in sympathetic postganglionic neurons and co-released with norepinephrine (NE) upon intense sympathetic activation, serving as a key co-transmitter. In the heart, NPY is the most abundant neuropeptide, primarily localized within sympathetic fibers innervating the coronary vasculature, endocardium, and myocardium. Furthermore, its presence within the ICG and select parasympathetic neurons provides a structural substrate for localized autonomic integration [47].

During high-intensity sympathetic arousal, the role of co-transmitters becomes increasingly prominent. Alongside NPY, sympathetic terminals can release adenosine triphosphate (ATP) and galanin. Whereas ATP undergoes rapid enzymatic degradation, NPY and galanin exhibit slow diffusion kinetics and extended half-lives, allowing them to serve as potent mediators of sustained autonomic regulation [48]. Such unique kinetic profiles position NPY as a central player in the long-term modulation of autonomic tone and the amplification of chronic pathophysiological effects.

At the level of interneuronal interaction, NPY serves as a pivotal mediator of the functional decoupling that characterizes sympathovagal imbalance. NPY released during heightened sympathetic activity acts upon presynaptic Y_2_ receptors located on cholinergic terminals. This triggers a potent inhibition of ACh release, thereby attenuating the vagal “braking effect” on cardiac chronotropy [49]. Indeed, the sustained suppression of vagally mediated bradycardia observed following intense sympathetic surges is critically dependent on NPY [49], identifying it not merely as an amplifier of sympathetic output but as a crucial molecular hub in autonomic dysregulation.

Beyond its indirect neuromodulation, recent evidence highlights the direct actions of NPY on cardiomyocytes. By binding to Y_1_ receptors on the sarcolemma, NPY profoundly remodels intracellular Ca^2+^ dynamics, characterized by increased Ca^2+^ transient amplitudes and abbreviated durations. Preclinical studies using ex vivo Langendorff-perfused rodent hearts have demonstrated that these alterations significantly heighten the propensity for ventricular arrhythmias [50]. Whether direct NPY-mediated modulation of human ventricular electrophysiology contributes meaningfully to clinical arrhythmogenesis remains an active area of investigation. Clinically, elevated coronary sinus NPY levels in patients with acute MI correlate with microvascular dysfunction, infarct expansion, and depressed ejection fraction, serving as an independent predictor of adverse outcomes in HF [51]. Furthermore, when high-dose β-blocker therapy fails to fully suppress sympathetic excitatory effects, NPY is implicated in driving the residual pro-arrhythmic burden that persists despite β-adrenergic blockade [52].

In summary, as a multifaceted co-transmitter, NPY provides a pivotal molecular bridge linking autonomic crosstalk, cardiomyocyte Ca^2+^ homeostasis, and arrhythmic vulnerability [3]. These findings suggest that pharmacological targeting of the NPY signaling axis or its receptors may offer a novel therapeutic avenue for antiarrhythmic intervention.

### Systemic neurohumoral nexus: The SAM–RAAS axis in arrhythmogenesis

Beyond the discrete molecular pathways of adrenergic and muscarinic signaling, the systemic orchestration of cardiac stability is governed by the synergistic interplay between the SAM axis and the RAAS [13]. This neurohumoral nexus serves as a critical bridge, mechanistically linking acute arrhythmic triggers to the chronic structural remodeling observed in diverse disease states.

Across various cardiovascular and endocrine disorders, chronic neurohumoral dysregulation functions as a central driver of both electrophysiological and structural remodeling, ultimately precipitating arrhythmias. In sinus tachycardia, acute stress triggers the SAM axis, inducing a profound and sustained elevation in catecholamine levels. Conversely, the RAAS is preferentially activated during states of relative hypovolemia (e.g., trauma or acute decompensated HF), where it exacerbates tachycardia through the presynaptic augmentation of sympathetic outflow [53]. In patients with HF, hypertension, or diabetes, persistent RAAS activation promotes atrial and ventricular fibrosis, establishing the structural substrate necessary for sustaining re-entrant circuits (human histological and imaging evidence). In the failing heart, this remodeling creates a nidus for micro-reentrant premature ventricular complexes (PVCs) and monomorphic ventricular tachycardia (VT), substantially increasing the risk of sudden cardiac death (SCD) [8]. Simultaneously, sustained RAAS activation synergizes with sympathetic signaling to derange repolarizing currents, such as *I*_Ks_, thereby heightening the risk of Torsades de Pointes (TdP) [54].

In long QT syndrome (LQTS), emotional or physical stress-induced sympathetic surges precipitate EADs. When compounded by electrolyte disturbances such as hypokalemia or hypomagnesemia (frequently secondary to diuretic use), these triggers further prolong the QT interval, predisposing patients to malignant ventricular arrhythmias [1]. Additionally, endocrine disorders are prominent drivers of persistent sinus tachycardia. For instance, pheochromocytoma causes abnormal catecholamine hypersecretion, while hyperthyroidism sensitizes the myocardium to catecholaminergic stimulation by up-regulating β_1_-AR expression and modulating ion channel kinetics [55].

Hyperthyroidism shortens the AVN refractory period, predisposing patients with concealed accessory pathways to paroxysmal supraventricular tachycardia (PSVT). In the presence of pre-excitation syndrome (Wolff–Parkinson–White syndrome), hyperthyroidism further abbreviates the accessory pathway refractory period, potentially leading to rapid ventricular conduction during AF and increasing the risk of degeneration into ventricular fibrillation (VF) [56]. Conversely, hypothyroidism slows metabolic rates to precipitate sinus bradycardia, while severe hyperkalemia depresses myocardial excitability, potentially inducing high-grade atrioventricular block [10]. In sick sinus syndrome (SSS), RAAS-mediated fibrosis within the SAN is the pathological hallmark of both isolated node dysfunction and the tachy-brady syndrome. Furthermore, chronic RAAS activation in hypertensive or dilated cardiomyopathies promotes progressive fibrosis of the conduction system, ultimately manifesting as intraventricular bundle branch blocks [57].

In summary, neurohumoral dysregulation acts as a fundamental nexus, tightly coupling acute electrophysiological instability with chronic structural remodeling. Across diverse pathological states, this imbalance perpetuates a deleterious feed-forward loop of remodeling–arrhythmia–remodeling, which significantly exacerbates the global burden of arrhythmic disease [13].

## The Neuroimmune Axis: Inflammation as a Convergent Pathway

The autonomic and humoral pathways discussed above do not operate in isolation; they interact bidirectionally with the immune system. This section illustrates the principle of convergent remodeling: Inflammatory mediators and neurotransmitters jointly influence ion channels, gap junctions, and fibrosis, thereby linking acute electrophysiological triggers with chronic structural remodeling. The discussion proceeds from the molecular mechanisms by which inflammatory mediators directly modulate cardiac electrical properties, to the bidirectional crosstalk between the autonomic nervous system and immune function, and finally to a critical appraisal of the cholinergic anti-inflammatory pathway as a therapeutic target in arrhythmias.

### Direct modulation of ion channels, gap junctions, and neuronal excitability by inflammatory mediators

Pro-inflammatory cytokines, notably tumor necrosis factor α (TNF-α) and interleukin-6 (IL-6), can directly augment SAN automaticity, thereby precipitating sinus tachycardia. Clinically, this manifests in acute hyper-inflammatory states, such as sepsis or severe COVID-19, in which a cytokine storm drives persistent tachycardia, as well as in chronic inflammatory disorders characterized by elevated resting heart rates. In myocarditis, localized inflammatory infiltration further irritates the SAN to induce tachyarrhythmic episodes [13].

Beyond automaticity, inflammatory mediators also profoundly disrupt myocardial repolarization and conduction through specific molecular actions on ion channels and gap junctions. TNF-α down-regulates the transient outward K^+^ current (*I*_to_) by suppressing voltage-gated K^+^ channels (K_v4.2_ and K_v4.3_) protein expression, and IL-6 increases *I*_CaL_, both of which contribute to action potential prolongation and triggered activity [58]. Furthermore, inflammatory mediators such as TNF-α also derange repolarization by inducing inducible nitric oxide synthase (iNOS) and elevating ROS production, which collectively suppress the human ether-à-go-go-related gene (hERG)-mediated rapid delayed rectifier K^+^ current (*I*_Kr_) [59]. The resultant prolongation of the APD increases the substrate for re-entrant arrhythmias. At the level of intercellular coupling, pro-inflammatory cytokines reduce the expression and membrane localization of Cx43, impairing gap junctional communication and slowing ventricular conduction velocity, thereby facilitating reentrant circuits [60]. Following MI, infiltrating macrophages further compromise inter-cardiomyocyte electrical coupling by modulating Cx43 proteostasis, and genetic ablation of GJA1 (gap junction protein α 1, encoding Cx43) exacerbates conduction disturbances and markedly increases arrhythmic susceptibility [10]. In patients with long QT syndrome type 2 (LQT2), pyrexia can exacerbate hERG channel dysfunction, leading to further QT interval prolongation and life-threatening arrhythmic events [1].

**Fig. 3.**
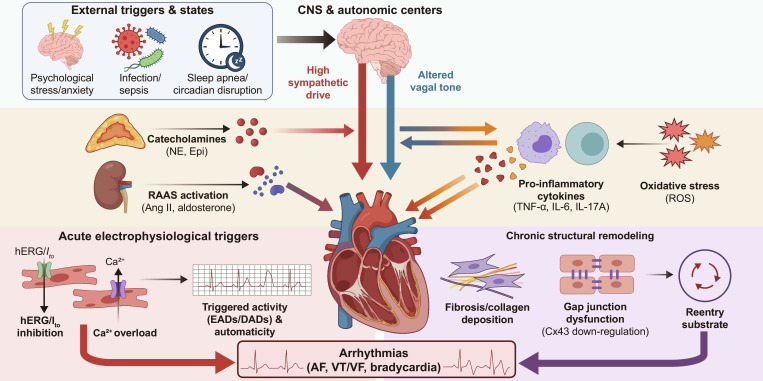
The integrated neuro-immune-humoral axis in the pathogenesis of cardiac arrhythmias. This schematic provides a hierarchical overview of the pathways linking external stressors to arrhythmogenic outcomes, as detailed in the accompanying text. (Top) Central initiation: external stressors (e.g., psychological stress, acute infection/sepsis, and sleep apnea/circadian disruption) activate central nervous system (CNS) autonomic centers. This results in a state of autonomic imbalance characterized by high sympathetic drive (red arrows) and altered (often reduced or dysfunctional) vagal tone (blue arrows). (Middle) Systemic mediators: The autonomic imbalance activates downstream systemic pathways. Elevated sympathetic outflow stimulates the adrenal glands to release catecholamines [norepinephrine (NE), epinephrine (Epi)] and the kidneys to activate the renin–angiotensin–aldosterone system (RAAS). Simultaneously, immune cells (e.g., macrophages and T cells) are activated to release pro-inflammatory cytokines [tumor necrosis factor α (TNF-α), interleukin-6 (IL-6), and IL-17A] and generate reactive oxygen species (ROS). Notably, bidirectional crosstalk exists, where inflammation further enhances sympathetic activity. (Bottom) Cardiac substrates and outcome: These neurohumoral and inflammatory mediators converge on the heart, driving both acute and chronic pro-arrhythmic mechanisms. Left (Acute): Mediators cause acute electrophysiological changes, such as ion channel dysfunction [e.g., human ether-à-go-go-related gene (hERG)/transient outward K^+^ current (*I*_to_) inhibition] and Ca^2+^ overload, leading to triggered activity [early afterdepolarizations (EADs)/delayed afterdepolarizations (DADs)] and enhanced automaticity. Right (Chronic): Long-term exposure leads to structural remodeling, characterized by fibrosis/collagen deposition and connexin 43 (Cx43) dysfunction, creating substrates for reentry. Outcome: The convergence of acute triggers and chronic substrates ultimately culminates in various clinical arrhythmias, including atrial fibrillation (AF), ventricular tachycardia (VT)/ventricular fibrillation (VF), and bradycardia. Red/orange arrows indicate pro-arrhythmic sympathetic/inflammatory pathways; blue arrows indicate parasympathetic pathways; purple arrows indicate structural pathological changes. Ang II, angiotensin II.

Beyond direct cardiomyocyte effects, inflammatory mediators modulate the excitability of autonomic neurons, thereby creating a feed-forward loop that perpetuates both inflammation and arrhythmogenesis. IL-1β and TNF-α enhance the firing rate of sympathetic neurons in the stellate ganglion and intracardiac ganglia, in part through the up-regulation of voltage-gated Na^+^ and Ca^2+^ channels [60,61]. Pro-inflammatory cytokines, including IL-6 and IL-17A, actively shape the neuro-cardiac interface by modulating neuronal excitability and neurotransmitter release. IL-17A, in particular, potentiates sympathetic overactivation through cyclin-dependent kinase 5 (CDK5)-dependent mechanism: Binding of IL-17A to its receptor on sympathetic neurons activates CDK5, which subsequently phosphorylates the pore-forming subunit of N-type voltage-gated Ca^2+^ channel (Ca_V2.2_), enhancing channel activity and facilitating Ca^2+^-dependent exocytosis of NE, thereby amplifying sympathetic outflow to the heart and increasing vulnerability to ventricular arrhythmias [62]. This neuronal sensitization amplifies sympathetic outflow to the heart, creating a self-reinforcing cycle (illustrated schematically in Fig. 3).

### Clinical manifestations of inflammation-driven arrhythmias

In the setting of atrial arrhythmias, inflammatory signaling disrupts atrial electrophysiology and structural integrity, thereby facilitating triggered activity and re-entrant circuits [37]. For instance, pro-inflammatory cytokines secreted by epicardial adipose tissue in patients with obesity or metabolic syndrome can infiltrate the adjacent atrial myocardium, exacerbating the risk of AF [54]. Postoperative AF (POAF), with an incidence of 30% to 50%, is largely driven by the acute inflammatory surge secondary to surgical trauma. Similarly, holiday heart syndrome underscores the arrhythmogenic potential of acute ethanol ingestion, where alcohol-induced inflammation and catecholaminergic activation converge to promote atrial arrhythmias [63]. Furthermore, during pyrexia, inflammatory mediators amplify the activity of ectopic foci, increasing the frequency of atrial premature beats (APBs) and subsequent palpitations [4].

Inflammation also directly compromises the electrical stability of the ventricles. In myocarditis, inflammatory foci within the myocardium can function as ectopic pacemakers, and the inflammatory infiltration characteristic of the acute phase of MI similarly destabilizes cardiac electrophysiology, heightening the risk of malignant ventricular arrhythmias such as VF [8].

Inflammatory cascades can also precipitate bradyarrhythmias. In myocarditis, direct inflammatory insult to the SAN may result in sinus bradycardia. In the advanced stages of severe sepsis or septic shock, a systemic inflammatory response often depresses conduction system function, leading to clinically significant bradycardia [64]. Chronic inflammation, associated with conditions such as sarcoidosis or autoimmune diseases, promotes fibrosis of the conduction system, potentially causing permanent atrioventricular block [56]. Acute myocarditis may transiently compromise SAN pacemaking and conduction through direct cell infiltration, tissue edema, and cytokine release. In contrast, sustained chronic inflammation drives progressive collagen deposition and structural remodeling within the SAN via prolonged exposure to pro-fibrotic mediators, such as TNF-α and transforming growth factor β1 (TGF-β1), ultimately leading to SSS [57].

Furthermore, inflammation impacts accessory pathways and intraventricular conduction. In patients with preexcitation syndrome, pyrexia can shorten the refractory period of the accessory pathway, augmenting the prominence of the delta wave and increasing the risk of rapid ventricular rates during AF [13]. Inflammatory cardiomyopathies, such as myocarditis and sarcoidosis, can induce intraventricular conduction block through direct injury to bundle branch tissue. Conversely, in cardiac amyloidosis, progressive conduction disturbances result from the structural disruption of conductive fibers caused by amyloid deposition [54].

### Bidirectional neuroimmune crosstalk and the cholinergic anti-inflammatory pathway

The relationship between autonomic signaling and inflammation is fundamentally bidirectional. While sympathetic overactivation promotes inflammation through β-adrenergic stimulation of immune cells, parasympathetic outflow exerts anti-inflammatory effects via the cholinergic anti-inflammatory pathway. Acetylcholine released from vagal efferents binds to α_7_ nicotinic acetylcholine receptors (α_7_nAChR) expressed on macrophages and other immune cells, inhibiting the nuclear translocation of nuclear factor κB (NF-κB) and suppressing the production of TNF-α, IL-1β, IL-6, and other pro-inflammatory cytokines [65–68]. α_7_nAChR is the essential receptor mediating this effect: VNS significantly attenuates endotoxin-induced serum TNF-α levels in wild-type mice but not in α_7_nAChR-deficient mice, and macrophages from α_7_nAChR knockout mice are refractory to cholinergic agonists [67]. Intracellularly, α_7_nAChR activation recruits the tyrosine kinase JAK2, leading to signal transducer and activator of transcription 3 (STAT3) phosphorylation, which blocks cytokine transcription by NF-κB, and also inhibits the NLR family pyrin domain containing 3 (NLRP3) inflammasome by preventing mitochondrial DNA release [68]. In experimental models of myocardial ischemia–reperfusion, VNS reduces myocardial cytokine levels, limits infarct size, and decreases arrhythmia susceptibility [65,67].

Conversely, pro-inflammatory cytokines act on vagal afferent terminals and central autonomic nuclei to modulate parasympathetic outflow. IL-1β, for instance, activates vagal afferents that project to the NTS, triggering reflex bradycardia and altering baroreflex sensitivity, while peripheral and central neuroinflammation contribute to sympatho-excitation and vagal withdrawal in HF [61]. This bidirectional crosstalk implies that autonomic imbalance not only is a cause of inflammation but also is shaped by the inflammatory milieu, establishing a dynamic interplay that influences arrhythmic vulnerability.

### Critical appraisal of the cholinergic anti-inflammatory pathway in arrhythmias

The strength of evidence supporting the cholinergic anti-inflammatory pathway as a therapeutic target in cardiac arrhythmias warrants careful evaluation. Preclinical studies have consistently demonstrated that VNS and α_7_nAChR agonists reduce inflammatory markers and improve electrical stability in animal models of MI, HF, and AF [65–67]. However, clinical evidence remains limited and mixed. While low-level VNS has shown promise in reducing AF burden in small pilot studies, the multicenter NECTAR-HF trial failed to demonstrate significant improvement in cardiac remodeling or functional capacity compared with optimized medical therapy, and the ANTHEM-HFrEF trial was terminated early [68]. The extent to which the beneficial effects of VNS are mediated specifically by anti-inflammatory mechanisms, as opposed to direct electrophysiological effects on the atria or modulation of autonomic tone, has not been rigorously dissected in human subjects. Moreover, the translational relevance of the cholinergic anti-inflammatory pathway may vary across disease states. In acute settings such as ischemia–reperfusion injury, vagal anti-inflammatory signaling appears protective, whereas in chronic HF, sustained parasympathetic withdrawal and altered muscarinic receptor expression may limit the efficacy of vagal stimulation [61]. Nicotinic agonists such as GTS-21 have shown anti-inflammatory effects in preclinical models and were well tolerated in phase I studies [67], but clinical translation to arrhythmia management remains unexplored. Future studies employing selective α_7_nAChR modulators, detailed immune phenotyping, and rigorous patient stratification will be necessary to determine whether this pathway can be harnessed for mechanism-guided antiarrhythmic therapy [68].

In parallel, angiotensin II, a central effector of the RAAS, not only amplifies inflammatory signaling but also drives myocardial fibrotic remodeling, creating a robust structural substrate for arrhythmogenesis [69].

Collectively, these findings underscore that arrhythmia initiation and progression arise from complex, multi-level interactions spanning neural, immune, endocrine, and structural domains. Accordingly, effective clinical management requires a paradigm shift beyond single-target interventions toward precision strategies that integrate neuromodulation with systemic anti-inflammatory and anti-remodeling therapies.

## Higher-Order Governance: Central Regulation and Circadian Rhythmicity

We now extend the regulatory hierarchy to the CNS and the circadian clock. These higher-order centers impose temporal dynamics on autonomic outflow, creating time-of-day-dependent windows of arrhythmic vulnerability. This section describes how stress, central autonomic commands, and circadian rhythms converge to shape arrhythmia patterns.

The CNS and the circadian system integrate to form a higher-order regulatory network governing cardiac electrophysiology. By modulating autonomic tone, myocardial ion channel kinetics, and the local electrophysiological substrate, this axis dictates arrhythmogenic triggers, onset patterns, and the temporal periodicity of arrhythmias [70]. The hierarchical interaction between these central regulatory hubs and the systemic neuro-humoral-immune cascades they initiate is integrated and illustrated in Fig. 3. Dysregulation within this hierarchy, arising from central pathologies, external stressors, or circadian misalignment, heightens susceptibility to various arrhythmias that frequently exhibit distinct chronobiological characteristics.

The CNS serves as the hierarchical regulatory hub orchestrating cardiac electrophysiology. By integrating afferent signals from the cerebral cortex, the limbic system (e.g., brain regions involved in emotional processing), and peripheral receptors within the medullary cardiovascular centers, the CNS finely tunes autonomic outflow to the peripheral nervous system [71]. This integrative hierarchy ensures precise modulation of chronotropy, dromotropy, and overall electrical stability, enabling the heart to adapt dynamically to physiological demands, environmental stressors, and emotional states while maintaining rhythmic integrity [3]. Stress-induced activation of higher-order brain centers skews the autonomic rheostat toward sympathetic dominance, potentiating NE release. NE, acting on β_1_-AR within the SAN, enhances pacemaker automaticity and precipitates sinus tachycardia. Consequently, the pathogenesis of sinus tachycardia, encompassing both psychogenic (e.g., anxiety or panic disorders) and neurogenic (e.g., stroke or traumatic brain injury) origins, is fundamentally rooted in CNS dysregulation [54]. Conversely, aberrant centrally mediated vagal reflexes can precipitate severe bradyarrhythmias. During vasovagal syncope, heightened vagal activation induces transient sinus bradycardia or asystole; similarly, the Cushing reflex, triggered by elevated intracranial pressure, drives robust central cardiac inhibition manifesting as sinus bradycardia and atrioventricular block [62]. In hearts with preexisting structural abnormalities, centrally driven sympathetic surges exacerbate local NE release, further destabilizing myocardial electrophysiological properties and lowering the arrhythmogenic threshold [72].

Circadian regulation of cardiac function is inextricably linked to the architecture of the CNS. The suprachiasmatic nucleus (SCN) of the hypothalamus, acting as the master circadian pacemaker, orchestrates the periodic modulation of autonomic tone, thereby governing cardiac rhythm and stability [2]. Physiological circadian rhythmicity entails a diurnal sympathetic bias to meet activity-induced metabolic demands, contrasted by nocturnal vagal dominance to facilitate myocardial recovery [14]. Chronodisruption significantly elevates arrhythmogenic risk; for instance, shift workers or individuals experiencing jet lag frequently exhibit a predisposition toward elevated resting heart rates and sinus tachycardia. Moreover, persistent nocturnal sinus tachycardia, a common feature of HF, diabetic neuropathy, and severe insomnia, serves as an adverse prognostic indicator reflecting sustained and aberrant sympathetic activation [73].

Circadian oscillations dictate the temporal periodicity of atrial arrhythmias: Diurnal sympathetic dominance predisposes individuals to APBs or atrial tachycardia (AT) via enhanced automaticity, whereas nocturnal vagal dominance promotes reentrant mechanisms conducive to AF [74]. Obstructive sleep apnea (OSA) serves as a paradigmatic model of combined circadian and autonomic dysregulation. During apneic episodes, hypoxia-driven parasympathetic activation is abruptly superseded by arousal-associated sympathetic surges. These profound autonomic fluctuations engender a highly arrhythmogenic milieu that triggers APBs, AT, and AF [63].

Nocturnal paroxysmal AF is intrinsically linked to hemodynamic loading, notably elevated atrial pressure or volume in the setting of HF or hypertension, and is primarily driven by nocturnal vagal predominance [75]. Under conditions of heightened nocturnal vagal tone, ACh released from parasympathetic terminals activates atrial M_2_ muscarinic receptors, stimulating *I*_KACh_ while concomitantly antagonizing *I*_CaL_ [76]. This synergistic ionic modulation truncates the atrial APD, consequently abbreviating the effective refractory period (ERP) and heightening its spatial dispersion. Such an electrophysiological milieu facilitates the induction and maintenance of re-entrant circuits, ultimately precipitating AF [4]. Upon morning awakening, a precipitous escalation in sympathetic outflow occurs, manifesting as the characteristic “morning surge”. This abrupt physiological transition exerts a disproportionate impact on patients with underlying structural heart disease, such as coronary artery disease or HF [77]. The concomitant catecholaminergic surge heightens myocardial excitability and triggered automaticity, thereby increasing the burden of PVCs and facilitating the transition to complex ventricular tachyarrhythmias [78]. Within proarrhythmic substrates, this transient electrical instability can degenerate into polymorphic VT or VF, markedly elevating the risk of SCD or malignant arrhythmic events [79].

In patients with LQTS, the incidence of life-threatening cardiovascular events exhibits a distinct circadian periodicity that is inextricably tied to genotype-specific triggers. In long QT syndrome type 1 (LQT1), arrhythmogenesis is predominantly precipitated by adrenergic surges during physical exertion or acute emotional stress. Conversely, LQT2 events frequently coincide with abrupt auditory-induced arousal, such as the ringing of an alarm clock during nocturnal sleep. In long QT syndrome type 3 (LQT3), arrhythmic clusters typically occur during nocturnal rest or bradycardic phases, underscoring a rate-dependent vulnerability modulated by the prevailing autonomic state [80]. Consequently, clinical management for LQT2 patients prioritizes the avoidance of acute arousal triggers, particularly high-decibel auditory stimuli, to mitigate the risk of centrally mediated sympathetic storms [13].

Thus, the synergy between the CNS and the circadian system dictates both the acute provocation of arrhythmias and the chronic exacerbation of underlying cardiac pathology. This multifaceted interplay ultimately defines a temporally dynamic window of vulnerability for arrhythmogenesis, where neural, temporal, and substrate-based factors converge to destabilize cardiac rhythm [2]. The chronobiological patterns of specific arrhythmia subtypes, including the morning peak of ventricular arrhythmias and the nocturnal predominance of vagally mediated AF, are summarized alongside their broader regulatory mechanisms in Table 1.

**Table 1.** Multimodal neural and systemic regulatory mechanisms across diverse clinical arrhythmic phenotypes

Arrhythmia type	Tachy/brady	Origin	Central and circadian	Peripheral nerves	Neurohumoral	Neuroimmune
Sinus tachycardia	Tachycardia	Sinus	Sympathetic central excitation (e.g., stress); more common during daytime [1]	NE release from cardiac sympathetic nerve endings [1,13]	HPA axis activation, increased circulating catecholamines [1]	Fever as a nonspecific trigger [1,13]
APBs/AT	Tachycardia	Atrial	Sympathetic excitation lowers threshold; more common during daytime [169]	Ectopic discharge from cardiac plexi (autonomic ganglia) can directly trigger events [170]	Increased circulating catecholamines raise atrial automaticity [171]	Atrial inflammation/fibrosis creates anatomical and electrophysiological substrate [172]
AF	Tachycardia	Atrial	Fluctuations in autonomic tone (sympathetic/parasympathetic) can trigger specific types of AF [173]	Core driver mechanism; autonomic ganglia in epicardial fat pads are key for AF initiation and maintenance [173]	Sympathetic and RAAS activation promote atrial electrical and structural remodeling [173]	Inflammatory cytokines cause abnormal atrial conduction, promoting AF initiation and persistence [173]
AVNRT/AVRT	Tachycardia	Supraventricular	Sympathetic excitation increases susceptibility; more common during daytime [174]	Vagal maneuvers (e.g., Valsalva) can effectively terminate acute episodes [174]	Hyperthyroidism is a significant risk factor; catecholamines lower induction threshold [174]	Acute inflammation/fever may act as a nonspecific trigger [174]
VPBs	Tachycardia	Ventricular	Sympathetic excitation (emotional stress) is the strongest trigger; exhibits a morning peak [175]	Areas of post-infarction denervation supersensitivity are prone to become VPB foci [176]	Core mechanism; acute catecholamine surge directly promotes VPBs; chronic RAAS activation leads to ventricular fibrosis, forming the substrate [176]	Acute myocarditis/ischemia directly creates VPB foci [176]
VT/VF	Tachycardia	Ventricular	Sympathetic storm is a direct, fatal trigger; exists a morning peak in risk (morning sudden death phenomenon) [177]	Hyperactivity of stellate ganglia and local cardiac sympathetic nerve sprouting significantly increase ventricular vulnerability [178]	Core substrate; RAAS is central to forming the substrate of ventricular fibrosis and malignant arrhythmias [179]	Acute myocardial ischemia/inflammation is a common acute and chronic substrate [1]
LQTS	Tachycardia	Other	Sudden sympathetic activation (e.g., startle) is the main trigger for LQT1/2; LQT2/3 have a nocturnal risk window [180]	The left stellate ganglion has a pronounced effect on repolarization dispersion [176]	Hypokalemia/hypomagnesemia directly inhibit K^+^ current, mimicking or worsening LQT2 [176]	Fever can affect channel function, altering LQT2/3 risk [176]
Sinus bradycardia	Bradycardia	Sinus	Physiological increase in vagal tone (e.g., sleep, athletes); more pronounced at night [181]	Reflexes like carotid sinus hypersensitivity can cause transient severe bradycardia [182]	Hypothyroidism and hyperkalemia directly suppress SAN function [1,13]	Acute myocarditis can directly damage the SAN [1]
SSS	Bradycardia	Sinus	Vagal excitation can worsen bradycardia; symptoms often more noticeable at night [1]	Abnormally sensitive to vagal reflexes, prone to long pauses [1,13]	Primary etiology; chronic RAAS activation leads to fibrosis and degeneration of the SAN and surrounding tissue [183]	Myocarditis can cause acute injury; chronic inflammation contributes to fibrosis [184]
Atrioventricular block	Bradycardia	Other	Vagal excitation (vasovagal reflex) causes transient block; physiological Wenckebach may occur at night [185]	Vagal nerve endings directly inhibit AVN function [186]	Drugs (e.g., β-blockers), hypothyroidism, and hyperkalemia are common reversible causes [1]	Important etiology; acute inflammation (e.g., myocarditis, Lyme disease) directly infiltrates and damages the conduction system [1,13]
Wolff–Parkinson–White syndrome	Other	Other	Sympathetic excitation increases susceptibility to AVRT; more common during daytime [13]	Vagal maneuvers can terminate AVRT episodes [174]	High-risk factor; hyperthyroidism significantly increases the risk of life-threatening AF with rapid conduction via the accessory pathway [187]	Fever may transiently affect conduction, playing a secondary role [1,13]
Intraventricular conduction block	Other	Other	-	-	Core association; RAAS activation is central to the fibrosis and structural block of the conduction system in the long term [188]	Important etiology; acute myocarditis, infiltrative diseases directly damage bundle branches [176]

NE, norepinephrine; HPA, hypothalamic–pituitary–adrenal; APBs, atrial premature beats; AT, atrial tachycardia; AF, atrial fibrillation; RAAS, renin–angiotensin–aldosterone system; AVNRT, atrioventricular nodal reentrant tachycardia; AVRT, atrioventricular reentrant tachycardia; VPBs, ventricular premature beats; VT, ventricular tachycardia; VF, ventricular fibrillation; LQTS, long QT syndrome; LQT1/2/3, long QT syndrome types 1, 2, and 3; SAN, sinoatrial node; SSS, sick sinus syndrome; AVN, atrioventricular node

## Maladaptive Neural Remodeling in Cardiac Disease

In pathological states such as MI and HF, the tightly regulated neural architecture becomes profoundly disrupted. This section demonstrates how regional denervation, denervation supersensitivity, and nerve sprouting create exaggerated spatial heterogeneity, and how these changes, together with altered transmitter release, fuel a self-reinforcing cycle of electrical instability. The mechanisms described here exemplify the interplay between spatial heterogeneity and convergent remodeling. Disease-induced remodeling of the cardiac nervous system illustrates the interplay between spatial heterogeneity and convergent remodeling that drives arrhythmogenesis in pathological states [10].

### Regional denervation and supersensitivity: Substrate for electrical instability

Denervated myocardial regions frequently manifest denervation supersensitivity, defined by a pathologically heightened electrophysiological and inotropic sensitivity to NE. This phenomenon has been robustly validated in experimental canine models of MI and subsequently corroborated in patients with ischemic cardiomyopathy undergoing electrophysiological study [11]. Notably, complementary evidence from Langendorff-perfused mouse hearts demonstrates that focal sympathetic hypoinnervation, independent of structural infarction, is sufficient to augment responsiveness to circulating catecholamines while blunting the efficacy of direct sympathetic nerve stimulation. Collectively, these findings underscore that the spatial heterogeneity of sympathetic innervation, per se, constitutes a potent pro-arrhythmic substrate [23].

**Fig. 4.**
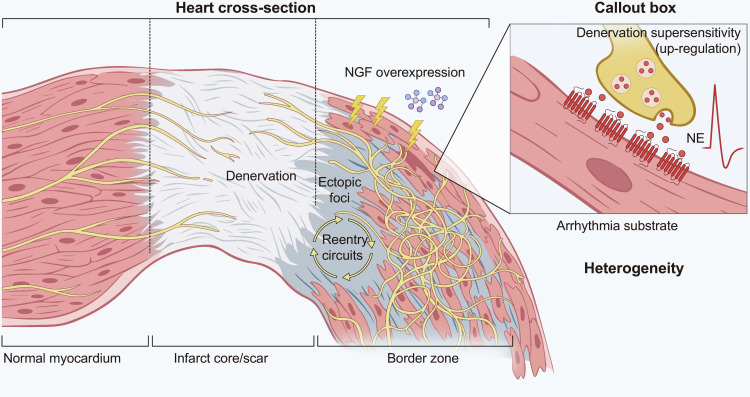
Pathological neural remodeling and spatial heterogeneity following MI. This schematic illustrates the regional changes in sympathetic innervation after MI and their contribution to the formation of an arrhythmogenic substrate, as discussed in the text. (Cross-section zones) The myocardium is divided into normal myocardium, the dense fibrous infarct scar/core, and the peri-infarct border zone. (Infarct core and Denervation): Within the scar tissue, nerve fibers undergo degeneration, leading to denervation. Cardiomyocytes in or adjacent to these denervated areas (Callout box) develop denervation supersensitivity, characterized by a compensatory up-regulation of surface β-AR, making them excessively responsive to even trace amounts of circulating catecholamines. (Border zone and Hyperinnervation) In the border zone, elevated levels of nerve growth factor (NGF), often driven by local inflammation, stimulate excessive and disorganized sprouting of new sympathetic nerve fibers (hyperinnervation). (Arrhythmic substrate): The juxtaposition of areas with sympathetic denervation/supersensitivity and areas with hyperinnervation creates significant spatial heterogeneity in electrical properties. The fibrotic scar creates anatomical blocks favoring reentry circuits, while abnormal neural activity in the border zone promotes the emergence of ectopic foci, together increasing susceptibility to malignant arrhythmias. NE, norepinephrine.

Interestingly, β-AR stimulation within the peri-infarct border zone has been shown to mitigate spatially heterogeneous alternans, thereby averting conduction block and the propagation of ectopic triggers [81]. Consequently, transmural MI and HF drive arrhythmogenesis through a synergistic dual-mechanism: first, by altering the myocardial substrate to create heterogeneous, slow-conducting scars that serve as an anatomical scaffold for reentry; and second, by remodeling the neural architecture to generate exaggerated and discordant adrenergic responses, which amplify the spatial dispersion of ventricular repolarization. The spatial interplay between denervated regions exhibiting supersensitivity and hyperinnervated border zones is depicted schematically in Fig. 4, which summarizes the key structural and functional consequences of post-infarction neural remodeling. The interplay between this structural remodeling and autonomic dysregulation is a central driver of VT in the failing and infarcted heart [81].

Preclinical models have established that post-MI arrhythmias typically originate from highly vulnerable niches within the infarct border zone. In clinical practice, the efficacy of catheter ablation for monomorphic VT, which targets border-zone corridors identified via high-density electroanatomical mapping, underscores the pivotal role of neuro-substrate remodeling in the genesis of ventricular tachyarrhythmias [82]. Furthermore, these mechanisms provide a robust mechanistic rationale for the clinical efficacy of β-blockers in significantly reducing mortality and arrhythmic burden in patients following MI or during HF.

### Neurotransmitter reprogramming and the impact of co-transmitter release

Beyond the primary adrenergic neurotransmitter NE, sympathetic postganglionic terminals co-release an array of neuropeptide modulators, most notably NPY and galanin. As discussed in detail in the Molecular Transduction and Neurohumoral Integration section, NPY acts as a potent pro-arrhythmic mediator during sustained sympathetic hyperactivation, both by inhibiting vagal ACh release and by directly modulating cardiomyocyte Ca^2+^ handling. Paralleling NPY, galanin is liberated during high-frequency sympathetic discharge. Under β-blockade, the pro-arrhythmic, sympathetically mediated blunting of vagal bradycardic responses can be partially mitigated by galanin receptor antagonism [83].

### Sympathetic hyperinnervation: Nerve sprouting and enhanced central drive

Pathological neural remodeling, specifically regional sympathetic hyperinnervation (nerve sprouting), acts as a primary catalyst for arrhythmias in various clinical contexts, notably HF, MI, and diet-induced obesity [84]. NGF stands as the central orchestrator of this process, driving axonal expansion through the activation of tropomyosin receptor kinase A (TrkA) signaling axis and subsequent phosphorylation of STAT3, as demonstrated in rodent models of MI and in vitro sympathetic neuron cultures. This process is further potentiated by gp130-activating cytokines, such as leukemia inhibitory factor (LIF) and cardiotrophin-1, which serve as indispensable co-factors for maximal outgrowth by facilitating the dual phosphorylation of STAT3 at both serine and tyrosine residues [12]. In the context of metabolic syndrome, adipokines like leptin synergize with NGF to exacerbate cardiac hyperinnervation [85]. Crucially, this structural expansion is intrinsically coupled with increased central sympathetic drive and a marked elevation in the excitability of postganglionic sympathetic neurons [86]. Morphological remodeling, such as the neuronal somata enlargement observed in patients with cardiomyopathy or canine models, correlates directly with heightened firing rates; similarly, in spinal cord injury models (e.g., T5 transection), expanded dendritic arbors and increased synaptic density provide the structural foundation for elevated cardiac sympathetic tone [87]. Ultimately, NGF-mediated retrograde TrkA signaling, which regulates synaptogenesis during development, is hijacked following injury to promote neuronal hypertrophy, synapse formation, and excessive excitability [88]. By transforming the CANS into a hyper-excitable and heterogeneously distributed network, this maladaptive remodeling provides the requisite substrate for neurogenic triggered activity, ensuring that even minor physiological stressors are amplified into catastrophic electrical instabilities. The molecular pathways driving this pathological nerve sprouting are illustrated in the context of post-infarction remodeling in Fig. 4.

### From neural dysregulation to clinical arrhythmias

The pathogenesis and progression of cardiac arrhythmias are dictated by a hierarchical regulatory landscape that encompasses the CNS, circadian rhythms, peripheral autonomic innervation, neurohumoral axes, and neuroimmune networks [7]. By converging upon the myocardium, these systems determine arrhythmogenic vulnerability and clinical trajectories through modulation of pacemaker automaticity, provocation of triggered activity, and consolidation of re-entrant circuits while simultaneously driving ion channel dysfunction and maladaptive structural remodeling [28]. Consequently, the broad clinical spectrum of arrhythmias, ranging from sinus tachycardia and AF to malignant ventricular tachyarrhythmias, is fundamentally governed by these convergent mechanisms [1]. In this setting, regulatory dysfunctions frequently coalesce into a self-perpetuating, pathological feed-forward loop; notably in HF, chronic sympathetic hyperactivation not only triggers acute arrhythmic events but also exacerbates long-term structural remodeling by recruiting sustained neuro-inflammatory responses. Clinically, pharmacological interventions such as β-blocker therapy are specifically employed to disrupt this maladaptive cycle [53]. Sympathetic and parasympathetic divisions homeostatically titrate cardiac rhythm and conduction. While balanced autonomic tone ensures electrical stability, any disruption of this equilibrium predisposes the heart to diverse arrhythmias [79]. The specific contributions of central, peripheral, neurohumoral, and neuroimmune factors to the initiation and maintenance of various arrhythmias are summarized in Table 1.

The clinical manifestations of autonomic imbalance span a wide spectrum, and inherited arrhythmia syndromes offer particularly instructive examples of how distinct autonomic profiles dictate specific arrhythmic phenotypes and therapeutic strategies. Two paradigmatic conditions—catecholaminergic polymorphic ventricular tachycardia (CPVT) and Brugada syndrome (BrS)—illustrate opposite ends of the autonomic spectrum: the former driven by sympathetic activation, the latter by vagal dominance. These disorders provide a unique window into the principles of mechanism-guided neuro-cardiac therapy, as their management is tailored not merely to the arrhythmia itself but to the specific autonomic trigger that provokes it.

CPVT serves as a primary model for understanding neuro-cardiac arrhythmias in the absence of structural heart disease [89]. The molecular pathophysiology of CPVT is centered on impaired calcium handling within cardiomyocytes, typically resulting from mutations in RyR2 or calsequestrin 2 (CASQ2) [90]. Under conditions of sympathetic arousal, PKA phosphorylates RyR2, significantly increasing the probability of spontaneous calcium release from the SR during diastole [91]. This diastolic calcium leak induces inward currents that generate DADs, serving as the trigger for life-threatening ventricular tachyarrhythmias. The management paradigm for CPVT is strategically focused on interrupting this sympathetic-driven process. First-line therapy involves nonselective β-blockers, particularly nadolol, due to its efficacy in blunting adrenergic responses [92]. For patients who remain symptomatic, flecainide is incorporated into the regimen, as it provides a synergistic effect by directly inhibiting RyR2-mediated calcium release and effectively suppressing exercise-induced arrhythmias [93]. In cases where pharmacological control is insufficient, LCSD remains a highly effective surgical intervention, as it directly reduces the adrenergic input to the ventricular myocardium and significantly lowers the incidence of SCD [94].

In contrast to CPVT, BrS represents an inherited arrhythmogenic disorder that is profoundly modulated by autonomic tone in the opposite direction. The arrhythmogenic substrate in BrS, typically localized to the RVOT, is highly sensitive to vagal dominance [95]. The characteristic electrocardiographic signature of BrS, marked by coved-type ST-segment elevation in the right precordial leads, is frequently exacerbated during sleep, rest, or post-prandial states when parasympathetic tone is elevated. Accordingly, life-threatening events, such as VF, predominantly occur at night [96]. Conversely, sympathetic activation or the administration of β-adrenergic agonists, such as isoproterenol, effectively normalizes the electrocardiographic pattern and suppresses electrical storms. This unique autonomic profile directly dictates the clinical management of BrS; during acute episodes, sympathomimetic agents are indicated, while drugs with vagolytic or anticholinergic properties, such as quinidine, are utilized chronically to counteract the arrhythmogenic vagal influence and reduce the recurrence of VF [97].

Together, CPVT and BrS illustrate a core principle of neuro-cardiac regulation: The autonomic profile of a given arrhythmia directly dictates its therapeutic strategy. While sympathetic-driven disorders such as CPVT respond to β-blockade and LCSD, vagal-dependent conditions such as BrS require sympathomimetic or vagolytic interventions. This divergence underscores the necessity of individualized, mechanism-guided approaches to arrhythmia management.

## Neuromodulatory Strategies: Linking Pathophysiology to Mechanism-Guided Therapy

**Fig. 5.**
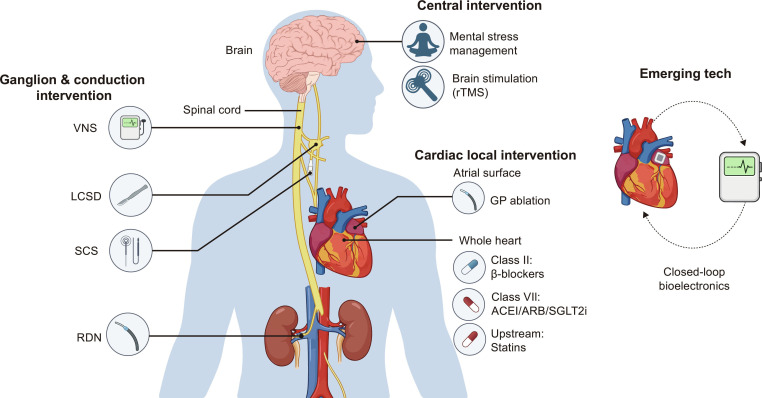
Overview of comprehensive neuromodulatory therapeutic strategies for arrhythmia management. (Central intervention) Targets the brain and higher centers through behavioral approaches like mental stress management or direct brain stimulation techniques to modulate autonomic outflow. (Ganglion & conduction intervention) Modulates autonomic signal transmission at the level of nerves and ganglia. This includes vagus nerve stimulation (VNS) to enhance parasympathetic tone, and procedures to reduce sympathetic drive such as left cardiac sympathetic denervation (LCSD) and spinal cord stimulation (SCS). (Cardiac local intervention) Directly targets the heart or upstream humoral modulators. This encompasses catheter ablation of atrial ganglionated plexi (GPs), renal denervation (RDN) to reduce central sympathetic drive, and pharmacological agents targeting β-AR, RAAS, and inflammation (statins). (Emerging tech) Highlights the development of closed-loop bioelectronic systems that use real-time physiological feedback from sensors to dynamically adjust neural stimulation parameters for precise arrhythmia control. rTMS, repetitive transcranial magnetic stimulation; ACEI, angiotensin-converting enzyme inhibitor; ARB, angiotensin II receptor blocker; SGLT2i, sodium–glucose cotransporter 2 inhibitor.

The mechanistic framework established in previous sections has direct therapeutic implications. Building upon the mechanistic principles outlined above, the contemporary management of arrhythmias has evolved into an integrated continuum that spans acute rhythm control and long-term substrate modification [98]. Whereas traditional antiarrhythmic strategies focused primarily on immediate rhythm stabilization, current approaches increasingly target the upstream drivers of electrical instability identified in preceding sections, including autonomic imbalance, neurohumoral overactivation, and chronic inflammation [99]. Within this expanded therapeutic landscape, neuromodulatory interventions occupy a central and rapidly growing role. An overview of the comprehensive neuromodulatory armamentarium, ranging from behavioral interventions to advanced bioelectronic systems, is provided in Fig. 5. This section first surveys the current pharmacological and nonpharmacological armamentarium for neuromodulation, then critically appraises the clinical evidence base for key interventions, examines the sources of patient heterogeneity that contribute to variable therapeutic responses, and finally discusses emerging technologies and future directions that promise individualized modulation of the neuro-cardiac axis.

### Pharmacological interventions targeting the neural substrate

The contemporary classification system for antiarrhythmic drugs, particularly its categories directly targeting neuromodulatory mechanisms, reveals a deeper therapeutic strategy: the treatment of arrhythmias through interventions on the autonomic nervous system, neurohumoral axes, and the intertwined immune-inflammatory network. Since the introduction of the Vaughan Williams scheme in the 1970s, which centered on ion channel modulation [100], classification systems have progressively broadened. The contemporary Oxford classification explicitly incorporates agents that act on neuromodulatory pathways, including modulators of autonomic tone, neurohumoral axes, and the intertwined immune-inflammatory network [15,100]. This expansion signifies a fundamental reorientation in therapeutic logic: moving beyond the suppression of electrical events toward modification of the arrhythmogenic neural substrate that sustains electrical instability [101]. The comprehensive classification of these agents, encompassing traditional ion channel blockers and modern upstream modulators, is summarized in Table 2.

**Table 2.** Modern classification of antiarrhythmic drugs [15]

Class		Mechanism/target	Representative drugs
Class 0		HCN channel blocker: SAN *I*_f_	Ivabradine
Class I	Ia	Na_v1.5_ blockers	Quinidine, ajmaline, disopyramide
	Ib	Na_v1.5_ blockers	Lidocaine, mexiletine
	Ic	Na_v1.5_ blockers	Propafenone, flecainide
	Id	*I*_NaL_ inhibitors	Ranolazine
Class II	IIa	β-Blockers	Nonselective β-inhibitors: carvedilol, propranolol, nadolol. Selective β_1_-AR inhibitors: atenolol, bisoprolol, betaxolol, celiprolol, esmolol, metoprolol
	IIb	β-Agonists	Isoproterenol
	IIc	M_2_ muscarinic blockers	Atropine, anisodamine, hyoscine, scopolamine
	IId	M_2_ muscarinic agonists	Carbachol, pilocarpine, methacholine, digoxin
	IIe	Adenosine receptor agonists	Adenosine, ATP
Class III	IIIa	Nonselective K^+^ channel blockers	Amiodarone, dronedarone
	IIIa	Selective hERG (*I*_Kr_) blockers	Sotalol, ibutilide, dofetilide, nifekalant
		K_v7.1_, K_v1.5_, K_v1.4_, K_v4.2_ blockers	Vernakalant, tedisamil
		Nonselective LTCC	Verapamil, diltiazem
	IIIb	*I*_KATP_ openers	Nicorandil, pinacidil
	IIIc	*I*_KACh_ blockers	Under clinical investigation (e.g., BMS-914392)
Class IV	IVa	Ca^2+^ channel blockers	Bepridil, verapamil, diltiazem
	IVb	SR RyR2-Ca^2+^ channel blockers	Flecainide, propafenone
Class V and VI		Mechano-sensitive channel and gap junction channel blockers	Under clinical investigation (e.g., carbenoxolone)
Class VII		Upstream targeting modulators: Intracellular signaling pathways	RAAS blockers (ACEI/ARB), MRA, SGLT2i, statins

HCN, hyperpolarization-activated cyclic nucleotide-gated channels; SAN, sinoatrial node; *I*_f_, funny current; Na_v1.5_, voltage-gated Na^+^ channel; *I*_NaL_, late Na^+^ current; β_1_-AR, β_1_-adrenergic receptor; ATP, adenosine triphosphate; hERG, human ether-à-go-go-related gene; *I*_Kr_, rapid delayed rectifier K^+^ current; K_v_, voltage-gated K^+^ channel; LTCC, L-type Ca^2+^ channel; *I*_KATP_, ATP-sensitive K^+^ current; *I*_KACh_, acetylcholine (ACh)-activated K^+^ current, SR, sarcoplasmic reticulum; RyR2, ryanodine receptor 2; RAAS, renin–angiotensin–aldosterone system; ACEI, angiotensin-converting enzyme inhibitor; ARB, angiotensin II receptor blocker; MRA, mineralocorticoid receptor antagonist; SGLT2i, sodium–glucose cotransporter 2 inhibitor

#### Class II agents: Modulating adrenergic G protein signaling

Class II agents directly target G protein-coupled receptors on cardiomyocytes, rapidly counteracting arrhythmias triggered by autonomic imbalance [102]. β-AR antagonists (e.g., propranolol, metoprolol, bisoprolol, and carvedilol) suppress sinus tachycardia and reduce atrial/ventricular ectopy by competitively inhibiting cardiac β_1_-AR. β-Blockade increases the threshold for VT/VF and provides well-established mortality benefit in MI and HF, with carvedilol conferring additional α-adrenergic antagonism that contributes to its antiarrhythmic profile [103]. Adenosine, by activating adenosine A_1_ receptors, produces transient AVN conduction suppression and remains a first-line agent for terminating PSVT dependent on AVN conduction [104]. M_2_ muscarinic receptor antagonists such as atropine block vagal inhibition, increasing SAN automaticity and AVN conduction velocity, and are primarily used for symptomatic bradyarrhythmia involving excessive parasympathetic activation [105].

#### Class VII modulators: Targeting upstream neuro-endocrine-immune pathways

Class VII agents encompass RAAS inhibitors, sodium–glucose cotransporter 2 inhibitor (SGLT2i), statins, and omega-3 fatty acids. These RAAS inhibitors, which include angiotensin-converting enzyme inhibitor (ACEI), angiotensin II receptor blocker (ARB), and mineralocorticoid receptor antagonist (MRA), attenuate myocardial fibrosis and adverse structural remodeling. By mitigating profibrotic and inflammatory signaling driven by angiotensin II and aldosterone, these agents reduce the incidence of AF and the risk of SCD in patients with HF and hypertension [106]. SGLT2is have emerged as pleiotropic cardiometabolic modulators: Large-scale trials (EMPEROR, DAPA-HF) report reduced cardiovascular mortality including sudden death, and emerging evidence suggests reduced AF incidence, likely mediated through attenuation of sympathetic activity, oxidative stress, and systemic inflammation [107]. Observational data and subgroup analyses suggest that statins and omega-3 fatty acids may confer indirect antiarrhythmic benefits through immunomodulatory and endothelial-stabilizing properties, particularly in peri-procedural settings, although the magnitude and consistency of benefit remain context-dependent [108].

#### Class 0 inhibitors: Indirect modulation via *I*_f_

Class 0 *I*_f_ inhibitors are represented by ivabradine, a highly selective *I*_f_ channel blocker. By selectively slowing spontaneous diastolic depolarization, it lowers heart rate while preserving broader autonomic signaling pathways, thereby occupying a unique position within the framework of neurohumoral regulation [109].

In HF accompanied by sinus tachycardia, persistent heart rate elevation increases myocardial oxygen demand, shortens diastolic coronary perfusion time, and may further amplify sympathetic drive, potentially through maladaptive baroreflex-mediated mechanisms. Ivabradine, by selectively reducing sinoatrial automaticity, not only lowers heart rate but also has been associated with improvements in HRV and reductions in circulating NE levels. These observations suggest that beyond pure rate control, *I*_f_ inhibition may exert indirect neuromodulatory effects by disrupting the feed-forward interaction between tachycardia and sympathetic activation [110]. Accordingly, in addition to improving outcomes in HF, ivabradine may represent a targeted therapeutic option for arrhythmias that are initiated or exacerbated by sustained tachycardia.

#### Other classes of modulators of contemporary classification system for antiarrhythmic drugs

Beyond these primary modulators, the Oxford framework incorporates a wide range of agents that interact with the neuro-cardiac axis across diverse mechanistic scales [15]. Class I and III agents, while traditionally viewed as ion channel blockers, serve to stabilize the myocardial substrate against the electrophysiological heterogeneity and repolarization dispersion amplified by autonomic signaling, including the specific blockade of *I*_KACh_ in the atria [15]. Class IV agents directly attenuate the intracellular Ca^2+^ overload and RyR2 instability driven by neurohumoral stress [15], while class V and VI modulators aim to preserve intercellular communication by regulating gap junction function within the inflammatory neuroimmune microenvironment [15]. Detailed information of Oxford classification is provided in Table 2.

#### Multifunctional agents: Synergizing neuromodulation and direct electrophysiological effects

Many antiarrhythmic drugs exert therapeutic effects through overlapping and complementary mechanisms, in which modulation of autonomic signaling operates in concert with direct electrophysiological actions [111]. Amiodarone exemplifies this integrated profile. Although traditionally categorized as a class III agent, its clinical efficacy extends beyond K^+^ channel blockade and reflects a broad spectrum of nonclassical effects, including noncompetitive antagonism of α-AR and β-AR, thereby conferring class II-like properties. In complex clinical settings such as AF or hemodynamically unstable VT, this combined electrophysiological and autonomic modulation contributes to rhythm stabilization, underscoring the therapeutic relevance of targeting both myocardial excitability and neurohumoral drive [112].

Carvedilol further illustrates the concept of integrated neuro-cardiac modulation. Beyond its β-adrenergic antagonism, this agent exhibits antioxidant and anti-apoptotic properties, which synergize with its upstream suppression of sympathetic signaling to confer broader cardioprotective effects [113]. Ranolazine provides another example of mechanistic convergence. By inhibiting the late sodium current, it stabilizes ventricular repolarization and attenuates repolarization heterogeneity. Emerging evidence suggests that ranolazine may also reduce spatial disparities in myocardial autonomic responses during ischemia, implying that part of its antiarrhythmic efficacy may derive from stabilization of the neuro-cardiac interface [114,115].

Ultimately, within the contemporary antiarrhythmic landscape, neuromodulatory pharmacotherapy has matured into a multi-faceted interventional strategy, spanning immediate receptor-level modulation to the long-term stabilization of the chronic disease substrate. A deeper understanding of how these agents influence the initiation and maintenance of arrhythmias by modulating autonomic tone, suppressing neurohumoral axis activity, and regulating the immune-inflammatory network not only provides a solid pathophysiological foundation for their rational clinical application but also points the way toward the future development of more precisely targeted therapies that modulate neuro-cardiac interactions.

### Nonpharmacological approaches: Surgical and device-based neuromodulation

Beyond pharmacological approaches, a spectrum of nonpharmacological interventions has expanded the therapeutic landscape of arrhythmia management by directly targeting neural structures and modulating autonomic function [102]. These strategies operate along the brain–heart and peripheral neuro-cardiac axes, aiming to restore neural homeostasis that underlies arrhythmia initiation and perpetuation [116]. Their scope spans ablative procedures, such as cardiac sympathetic denervation for refractory VT, neuromodulatory stimulation techniques, including transcutaneous auricular VNS for AF prevention, and selected behavioral interventions. Collectively, these approaches underscore the growing recognition that arrhythmic disease may be amenable to targeted modulation of neuro-cardiac interactions [117,118].

#### Ablative and interventional neuromodulation: RDN, LCSD, and GP ablation

RDN employs catheter-based ablation of peri-arterial renal sympathetic fibers to reduce central sympathetic outflow to the heart [16,119]. While its antihypertensive efficacy has been demonstrated in sham-controlled trials (SPYRAL HTN-OFF MED, RADIANCE-HTN SOLO), evidence for direct antiarrhythmic benefit, particularly as an AF therapy, remains inconsistent and limited to small observational studies and post hoc analyses [120,121]. A critical appraisal of the translational gaps will be discussed later.

Cardiac GP ablation targets epicardial autonomic integration hubs to attenuate maladaptive autonomic input to the atria [122,123]. Adjunctive GP ablation alongside pulmonary vein isolation (PVI) has been investigated for reducing AF recurrence, with early nonrandomized studies suggesting improved rhythm control [17,124]. However, the randomized AFACT trial found no improvement in sinus rhythm maintenance with additional GP ablation in persistent AF while paradoxically reporting increased iatrogenic AT [125]. The clinical role of GP ablation as an adjunctive strategy remains uncertain and will be further introduced later.

In AF, particularly paroxysmal AF, aberrant activity within these plexi has been implicated as both a trigger and a modulator of arrhythmic maintenance [123,126]. Adjunctive GP ablation, typically performed alongside PVI, aims to attenuate maladaptive autonomic input to the atria. Guided by electrophysiological mapping techniques, targeted ablation of major plexi seeks to suppress autonomically mediated triggers and reduce the stability of re-entrant circuits [17,124]. By dampening excessive or heterogeneous autonomic influence, this strategy has been associated with improved procedural efficacy and greater long-term rhythm control in selected patients [124,125]. Such an approach may be particularly relevant in individuals whose AF exhibits marked sensitivity to autonomic fluctuations, including episodes linked to nocturnal vagal predominance or heightened daytime sympathetic activity [17,122].

LCSD represents a surgical strategy aimed at sustained attenuation of ventricular sympathetic input. The procedure typically involves thoracoscopic resection of the lower portion of the left stellate ganglion and upper thoracic sympathetic chain [18]. Its principal mechanism is the selective and durable reduction of adrenergic drive to the ventricles, particularly the left ventricle, thereby increasing the threshold for VF while exerting minimal effects on resting heart rate [8]. LCSD constitutes a cornerstone therapy in selected inherited arrhythmia syndromes. It is well established in the prevention of SCD in catecholaminergic polymorphic VT and serves as an adjunctive or rescue strategy in patients with LQTS who remain symptomatic despite, or are intolerant of, β-adrenergic blockade [127]. In the setting of post-MI electrical storm or recurrent implantable cardioverter-defibrillator (ICD) shocks, LCSD has also been employed to suppress refractory ventricular arrhythmias by directly interrupting pathologically amplified sympathetic signaling [18]. In CPVT, LCSD directly addresses the pathophysiological cascade: By interrupting the sympathetic efferent limb that triggers PKA-mediated RyR2 hyperphosphorylation and diastolic Ca^2+^ leak, it removes the upstream driver of DAD-mediated triggered activity.

#### Bioelectronic stimulation: VNS, SCS, and baroreceptor activation

In contrast to ablative approaches, electrical stimulation-based neuromodulation seeks to regulate, rather than disrupt, autonomic neural circuits. These strategies modulate the activity of targeted neural pathways through controlled electrical stimulation, with the aim of restoring dynamic autonomic equilibrium. Notably, their effects are reversible and titratable, allowing adaptive adjustment of neural output without permanent alteration of neural anatomy.

Spinal cord stimulation (SCS) represents a neuromodulatory strategy that targets central autonomic pathways through epidural electrical stimulation of the thoracic spinal cord. By engaging inhibitory circuits within the dorsal horn, SCS is thought to attenuate sympathetic outflow from brainstem cardiovascular centers while potentially augmenting vagal activity [128]. Traditionally recognized for its anti-ischemic and sympathoinhibitory effects, SCS is increasingly being explored as a therapeutic option in arrhythmic disease [19]. Emerging investigations have examined its application in ventricular arrhythmias associated with refractory angina, as well as its potential role as an adjunctive strategy for managing electrical storm in patients with recurrent ICD therapies [129]. By reducing the overall sympathetic tone to the heart, SCS helps stabilize myocardial electrical activity, thereby potentially reducing the incidence of malignant arrhythmias [19,129]. To date, clinical evidence consists primarily of case series and small uncontrolled studies; large randomized trials are needed to establish efficacy and define optimal patient selection.

VNS encompasses both implantable and noninvasive (transcutaneous auricular) modalities that augment parasympathetic tone and rebalance autonomic control [20,130]. While preclinical models consistently demonstrate antiarrhythmic and anti-inflammatory effects, clinical translation has encountered significant obstacles: The INOVATE-HF trial failed to demonstrate mortality or hospitalization benefit in HF, and NECTAR-HF showed no improvement in cardiac remodeling. Small studies in paroxysmal AF (e.g., TREAT AF) suggest potential for AF burden reduction, but large randomized controlled trials (RCTs) with arrhythmia-specific primary endpoints are lacking [131,132].

Carotid sinus stimulation, also referred to as baroreceptor activation therapy, involves electrical activation of carotid sinus baroreceptors through an implanted device. By mimicking the afferent signaling associated with elevated arterial pressure, this intervention engages the baroreflex arc, leading to suppression of central sympathetic outflow and augmentation of vagal activity [133,134]. Although primarily developed for resistant hypertension, the sustained reduction in heart rate and global sympathetic tone achieved with this approach provides a mechanistic rationale for its potential application in selected arrhythmic conditions. In particular, baroreflex activation has been proposed as a strategy for sympathetic-dependent tachyarrhythmias or as an adjunctive modality for rate control in AF [135]. However, its antiarrhythmic efficacy remains under ongoing clinical evaluation [136].

#### Lifestyle and behavioral interventions: HRV biofeedback and mind–body practices

Beyond device-based neuromodulation, behavioral interventions aim to restore neural homeostasis by reshaping autonomic regulatory patterns at the cortical and systemic levels over time. HRV biofeedback and structured breathing training represent prototypical approaches within this category. By guiding individuals toward slow, diaphragmatic breathing at resonance frequencies (typically 4 to 6 breaths per minute), these techniques enhance respiratory sinus arrhythmia, a surrogate marker of vagal tone, and improve baroreflex sensitivity [21,137]. With regular practice, such interventions may increase baseline parasympathetic activity and promote more stable autonomic regulation [21]. Clinically, this method has been incorporated into comprehensive management plans for patients with paroxysmal AF and symptomatic ventricular premature beats (VPBs) [138]. They appear particularly relevant for patients in whom arrhythmic episodes are closely linked to emotional stress or anxiety, and have been associated with reductions in symptom burden and, in some cases, decreased reliance on antiarrhythmic pharmacotherapy [137].

Mind–body practices, including yoga, meditation, and Tai Chi, represent nonpharmacological strategies that integrate controlled movement, breathing, and attentional focus to evoke a coordinated relaxation response. Through engagement of central regulatory circuits, these approaches are thought to attenuate chronic activation of the hypothalamic–pituitary–adrenal (HPA) axis and sympathetic nervous system, thereby reducing circulating stress mediators such as cortisol and catecholamines while enhancing parasympathetic activity [139]. RCTs have reported that structured yoga interventions can reduce episode frequency and duration in patients with paroxysmal AF and improve quality-of-life metrics [22]. These findings support the concept that “top-down” modulation from higher cortical centers may favorably influence autonomic balance and, in turn, stabilize the cardiac electrophysiological substrate [21,139].

Sustained, moderate-intensity aerobic exercise represents a foundational strategy for long-term autonomic remodeling [140]. Beyond promoting adaptive structural and functional cardiac remodeling, regular exercise enhances vagal cardiac control, lowers resting heart rate, and attenuates baseline sympathetic tone [116]. In the secondary prevention of AF, particularly following catheter ablation, exercise-based rehabilitation has been shown to significantly lower the risk of AF recurrence [141]. This benefit stems not only from improved hemodynamics but also, more profoundly, from exercise training optimizing the autonomic nervous system’s dynamic regulatory capacity over the heart, thereby enhancing its electrical stability under stress [140].

### Critical appraisal and evidence grading of current neuromodulatory strategies

While the therapeutic rationale for neuromodulation is grounded in compelling mechanistic principles, its translation into clinical practice has yielded mixed and, in several notable cases, neutral or negative results. We critically appraise the clinical evidence for key interventional approaches, highlight discordant trial outcomes, and grade the current strength of evidence to provide a transparent framework for distinguishing interventions with mature clinical validation from those that remain experimental or transitional.

#### RDN: Inconsistent outcomes in AF

RDN has a robust physiological basis as an antiarrhythmic intervention: Ablation of peri-arterial renal sympathetic fibers reduces central sympathetic outflow, thereby attenuating systemic sympathetic tone—a key driver of atrial arrhythmogenesis [16,119]. Early nonrandomized studies suggested that RDN combined with PVI reduced AF recurrence compared with PVI alone. However, subsequent sham-controlled trials have painted a more complex picture. The SYMPLICITY HTN-3 trial, the first large sham-controlled trial, failed to demonstrate a significant blood pressure-lowering effect of RDN, which initially cast doubt on its application for arrhythmia management [119]. Although subsequent trials like SPYRAL and RADIANCE have shown modest efficacy, the initial failure highlights the critical importance of technical factors, such as the completeness of circumferential ablation, and the confounding effects of medication adherence [142,143]. The SPYRAL HTN-OFF MED and RADIANCE-HTN SOLO trials confirmed the antihypertensive efficacy of RDN, yet AF-related outcomes in these studies were either post hoc analyses or secondary endpoints, limiting their evidentiary weight for arrhythmia-specific indications [142]. A fundamental question remains unresolved: Is any potential antiarrhythmic benefit of RDN independent of blood pressure reduction, or is it merely an epiphenomenon of antihypertensive efficacy? Currently, no large-scale randomized sham-controlled trial with AF as the primary endpoint has been completed. Furthermore, procedural heterogeneity—including ablation modality (radiofrequency versus ultrasound), completeness of circumferential ablation, and patient selection criteria—varies substantially across studies, complicating cross-trial interpretation. Until dedicated trials with arrhythmia-specific primary endpoints are available, RDN should not be recommended as a standalone therapy for AF.

#### VNS: Translational hurdles and evidence gaps in arrhythmia

VNS has consistently demonstrated cardioprotective and antiarrhythmic effects in preclinical models, yet its clinical translation has encountered formidable obstacles. The INOVATE-HF trial failed to demonstrate a reduction in the composite endpoint of death or HF hospitalization with implantable VNS in patients with HF with reduced ejection fraction (HFrEF) [144]. This discrepancy between promising preclinical data and neutral clinical outcomes is likely attributable to insufficient neural dosing, where stimulation parameters failed to consistently engage the necessary fibers, and the high degree of interpatient variability in autonomic responsiveness. Similarly, the NECTAR-HF trial showed no significant improvement in left ventricular remodeling. These neutral outcomes have prompted critical discussion regarding “neural dosing”—whether stimulation parameters were sufficient to effectively engage vagal efferent fibers, and whether interpatient variability in baseline autonomic function contributed to heterogeneous responses. In the arrhythmia domain, small studies such as TREAT AF have suggested that low-level transcutaneous VNS may reduce paroxysmal AF burden [131,132]. However, these studies were modest in size and duration, and large RCTs with arrhythmia-specific primary endpoints are absent. Furthermore, the AFACT trial paradoxically reported that adjunctive GP ablation—which partially interrupts vagal input to the atria—increased the incidence of iatrogenic AT, highlighting the complex and sometimes unpredictable consequences of modulating atrial parasympathetic innervation [125]. At present, stimulation parameters, target populations, and efficacy endpoints for VNS in arrhythmia management remain unstandardized, and both the invasiveness and cost-effectiveness of implantable devices warrant careful consideration. Future investigation should prioritize rigorous patient stratification, potentially guided by autonomic function testing, and the development of closed-loop adaptive stimulation algorithms.

#### GP ablation: Uncertain evidence as an adjunct to PVI

The GPs embedded within epicardial fat pads serve as critical integration hubs for cardiac autonomic signaling, and their ablation has been proposed to suppress autonomically mediated triggers and modulate the atrial substrate that sustains AF [122,123]. Early nonrandomized studies reported that adjunctive GP ablation alongside PVI reduced AF recurrence in patients with paroxysmal AF [17,124]. However, the randomized AFACT trial, which evaluated thoracoscopic PVI with or without GP ablation in patients with persistent AF, did not demonstrate improved sinus rhythm maintenance at 1 year. Notably, GP ablation was associated with a significantly increased incidence of macro-reentrant AT [125]. These findings raise fundamental questions regarding the clinical value of GP ablation. Possible explanations include inadequate or excessive ablation extent, discordance between anatomical GP localization and functional mapping, and a relative sympathetic predominance following vagal denervation. The optimal target population (paroxysmal versus persistent AF), ablation endpoint (anatomical versus high-frequency stimulation-guided), and long-term benefit-to-risk ratio remain undefined. Given the available evidence, GP ablation should not be considered a routine adjunctive strategy and warrants further investigation in carefully designed randomized trials.

#### SCS: Inconsistent clinical results and unstandardized protocols

Recent studies on SCS have yielded inconsistent results, underscoring the lack of standardized stimulation protocols and the challenges in patient stratification. While preclinical studies consistently demonstrate that thoracic SCS reduces ventricular arrhythmias during acute ischemia by attenuating sympathetic outflow and modulating spinal interneuron activity [128], clinical evidence remains limited to case series and small uncontrolled studies. No large-scale randomized trial has been completed to establish efficacy or define optimal patient selection. Furthermore, stimulation parameters (frequency, intensity, electrode positioning) vary widely across reports, and the optimal neural target within the spinal dorsal horn for cardioprotection has not been systematically defined [19]. The invasive nature of epidural electrode implantation and the associated risks of lead migration, infection, and hardware malfunction further complicate the risk–benefit assessment.

Beyond the areas discussed above, additional translational challenges warrant attention. The CANTOS trial established that IL-1β inhibition reduces major adverse cardiovascular events but did not demonstrate a clear antiarrhythmic benefit [145], suggesting that systemic anti-inflammatory strategies may be insufficient to address localized neuroimmune remodeling within the heart.

Collectively, this critical appraisal reveals a notable asymmetry between translational enthusiasm and the strength of available clinical evidence for several interventional neuromodulation approaches. Among the strategies discussed, LCSD is supported by the most mature evidence base, with decades of observational data and consensus guideline recommendations for specific inherited arrhythmia syndromes. β-Adrenergic blockade remains the foundational pharmacological neuromodulation strategy, with unequivocal mortality benefit demonstrated in large randomized trials across MI and HF populations. In contrast, RDN, VNS, SCS, and GP ablation occupy a spectrum of clinical uncertainty: While their mechanistic rationale is compelling and preclinical data are encouraging, each lacks definitive evidence from adequately powered RCTs with arrhythmia-specific primary endpoints. The gap between preclinical promise and clinical validation is particularly pronounced for VNS, where 2 large HF trials (INOVATE-HF, NECTAR-HF) failed to meet their primary endpoints, and for GP ablation, where the sole randomized trial (AFACT) was negative. In this context, we caution that clinical adoption of these interventional modalities should proceed within the framework of ongoing clinical trials and registry-based evidence collection, rather than as routine or first-line strategies. The thoughtful matching of therapeutic enthusiasm to evidentiary rigor is essential to ensure that the promise of neuro-cardiac neuromodulation translates into meaningful clinical benefit. A graded overview of the clinical readiness of major neuromodulatory strategies is provided in Table 3.

**Table 3.** Summary of key neutral or mixed clinical trials in neuromodulation

Trial name	Intervention	Primary result	Potential reasons for neutral/mixed outcomes	References
SYMPLICITY HTN-3	RDN	Neutral (blood pressure reduction)	Incomplete nerve ablation; variability in procedural technique; medication changes	[189]
INOVATE-HF	VNS	Neutral (HF outcomes)	Insufficient neural recruitment; nonstandardized stimulation parameters; patient heterogeneity	[144]
CANTOS	Canakinumab (IL-1β inhibition)	Neutral (arrhythmia)	Systemic vs. localized inflammation; complexity of the bidirectional neuroimmune axis	[145]
NECTAR-HF	VNS	Neutral (LV remodeling)	Sub-threshold stimulation levels; potential off-target effects	[190]
Recent SCS studies	SCS	Mixed (VT reduction)	Small cohort sizes; lack of optimized or closed-loop stimulation protocols	[191]

RDN, renal denervation; VNS, vagus nerve stimulation; HF, heart failure; IL-1β, interleukin-1β; LV, left ventricle; SCS, spinal cord stimulation; VT, ventricular tachycardia

#### Evidence grading of neuromodulatory therapies for arrhythmias

Table 4 summarizes the evidence level, clinical readiness, and human validation status of each major strategy discussed in this review. Interventions are classified along a spectrum from established therapies with definitive RCT support to experimental platforms still confined to preclinical models. This graded framework is intended to guide clinical decision-making and inform the design of future trials by clearly delineating where evidence is mature and where it remains preliminary.

**Table 4.** Evidence grading and clinical readiness of neuromodulatory therapies for arrhythmias

Therapeutic strategy	Target arrhythmia	Grading of evidence	Current clinical readiness and human validation	References
β-AR blockers	LQTS, CPVT, HF, AF	High (large RCTs, meta-analyses)	Established. Class I guideline recommendation for most sympathetically driven arrhythmias.	[176]
LCSD	Refractory LQTS, CPVT	High (large clinical registries, multi-center cohorts)	Established. Guideline-directed surgical option for high-risk inherited arrhythmias failing medical therapy.	[192,193]
GP ablation	AF	Moderate to high (RCTs)	Clinically applied. Used primarily as an adjunctive strategy alongside PVI.	[173]
RDN	AF with concurrent hypertension	Moderate (mixed RCTs)	Transitional. Human validation shows mixed efficacy (e.g., SYMPLICITY trials); modest arrhythmic benefits observed in specific subgroups.	[189,194]
VNS	HF, ventricular arrhythmias	Moderate (preclinical robust; mixed/negative RCTs)	Transitional. Extensive animal validation but large human RCTs (e.g., INOVATE-HF) failed to show primary endpoint benefits.	[144,195]
SCS	Refractory VT, angina	Low to moderate (phase I/II, small cohorts)	Transitional. Limited human data with variable antiarrhythmic efficacy; stimulation parameters remain unstandardized.	[196]
Optogenetics/chemogenetics	Spatiotemporal neural control	Preclinical only	Experimental. Highly mechanistic; restricted to transgenic animal models.	[197]
Gene therapy (e.g., viral vectors targeting stellate ganglia)	Sympathetic hyperactivity	Preclinical only	Experimental. Validated in canine/porcine models; major hurdles remain regarding delivery and off-target effects in humans.	[198]
Nanomaterial bioelectronics	Closed-loop neuromodulation	Preclinical only	Experimental. Early-stage testing in rodents; human biocompatibility and long-term stability are unknown.	[199]

β-AR; β-adrenergic receptor; LQTS, long QT syndrome; CPVT, catecholaminergic polymorphic ventricular tachycardia; HF, heart failure; AF, atrial fibrillation; RCTs, randomized controlled trials; LCSD, left cardiac sympathetic denervation; GPs, ganglionated plexi; PVI, pulmonary vein isolation; RDN, renal denervation; VNS, vagus nerve stimulation; SCS, spinal cord stimulation

### Sources of heterogeneity in the neuro-cardiac axis: Implications for precision neuromodulation

The inconsistent and frequently discordant outcomes documented in the preceding section highlight a fundamental challenge in neuromodulation: the assumption that a uniform intervention will produce consistent benefit across diverse patient populations. This assumption is belied by the inherent heterogeneity of the neuro-cardiac axis. The neuro-cardiac axis is not a uniform regulatory system; rather, its functional properties and arrhythmic manifestations are profoundly shaped by age, sex, genetic background, comorbidities, and concomitant medications. Acknowledging these sources of heterogeneity is essential for the rational implementation of precision neuromodulatory strategies.

#### Age

Aging is associated with progressive structural and functional changes in cardiac autonomic regulation. This process is typically characterized by a gradual decline in vagal tone and an increase in systemic inflammatory activity, often termed “inflammaging”, which heightens cardiac sensitivity to sympathetic surges [146]. These shifts include diminished parasympathetic tone, increased basal sympathetic activity, and blunted β-adrenergic responsiveness due to receptor down-regulation [147]. Such age-related alterations reduce HRV and impair baroreflex sensitivity, thereby narrowing the dynamic range of autonomic control. Consequently, older patients may exhibit altered responses to both pharmacological β-blockade and device-based neuromodulation. For example, the efficacy of β-blockers in reducing arrhythmic events may be attenuated in elderly patients, while the bradycardic and hypotensive side effects of both β-blockers and VNS may be amplified. The age-dependent decline in parasympathetic reserve also has implications for VNS: The capacity for vagally mediated cardioprotection may be reduced, suggesting that VNS efficacy observed in younger animal models may not fully translate to older human populations.

#### Sex hormones

Sex hormones exert significant modulatory effects on autonomic regulation and cardiac electrophysiology. Estrogen enhances parasympathetic tone and confers relative protection against sympathetically driven arrhythmias in premenopausal women, whereas testosterone tends to promote sympathetic dominance. Correspondingly, there are inherent differences in basal autonomic tone: Healthy females typically exhibit higher resting parasympathetic (vagal) tone, while males tend toward higher sympathetic tone [148]. These differences influence the epidemiology of arrhythmias; for example, the sympathetic predominance in males is associated with a higher overall incidence of AF [149].

Regarding the electrophysiological baseline, females typically have a longer baseline QT interval than males. This difference primarily arises from the selective modulation of cardiac delayed rectifier K^+^ currents: Estrogen tends to inhibit *I*_Kr_ and *I*_Ks_ currents, whereas testosterone has a promoting effect, leading to a relatively lower ventricular repolarization reserve in females [150]. Clinically, while women exhibit higher baseline HRV and a lower incidence of SCD compared with age-matched men, they are more prone to significant QT interval prolongation when facing intense sympathetic surges (such as stress states) or using drugs that affect ion channels. Consequently, they are disproportionately affected by malignant events like drug-induced TdP [151].

These sex-specific physiological baselines have direct implications for neuromodulation. The higher vagal baseline in females may act as a buffer against sympathetically driven triggers, but it may also modify their response curves to interventions like VNS [152]. Therefore, women may derive differential benefit from parasympathetic-enhancing strategies (e.g., VNS and HRV biofeedback), making sex-based stratification vital for optimizing therapeutic outcomes when implementing targeted treatment strategies [152]. Furthermore, pregnancy and the postpartum period represent dynamic states of autonomic and hormonal flux that can unmask or exacerbate latent pro-arrhythmic substrates—as exemplified by the increased risk of cardiac events in women with LQT2 during the postpartum period [153].

#### Comorbidities

Chronic diseases, particularly metabolic comorbidities like diabetes mellitus and obesity, substantially alter autonomic balance and may confound neuromodulatory interventions. Diabetes with autonomic neuropathy is characterized by initial parasympathetic withdrawal followed by progressive sympathetic denervation, creating a state of functional denervation supersensitivity and impaired baroreflex function [154]. The resulting loss of physiological HRV blunts the prognostic utility of HRV-based risk stratification and may attenuate the therapeutic efficacy of VNS, as the efferent neural pathway may be damaged. Concurrently, obesity is associated with sympathetic overactivation driven by hyperleptinemia, insulin resistance, and OSA, all of which contribute to atrial and ventricular arrhythmogenesis [155]. Furthermore, obesity-associated expansion of epicardial adipose tissue serves as a local reservoir for pro-inflammatory cytokines and adipokines [156]. These local mediators directly influence the ICNS and atrial electrophysiology. While the enhanced sympathetic drive in obesity may theoretically increase the therapeutic window for sympathetic inhibition (e.g., β-blockade and RDN), the combination of local inflammation and advanced structural remodeling may simultaneously render the atrial substrate more refractory to standard rhythm control strategies, including ablation and stimulation-based therapies [156].

#### Medication interactions

Many commonly prescribed medications modulate autonomic tone or cardiac electrophysiology and may interact with neuromodulatory therapies. Tricyclic antidepressants, antipsychotics, and certain antiemetics prolong the QT interval through *I*_Kr_ blockade, an effect that may be exacerbated by agents that reduce sympathetic drive and slow heart rate. Conversely, medications with sympathomimetic effects (e.g., bronchodilators, decongestants, and stimulants) may counteract β-adrenergic blockade and increase arrhythmic susceptibility [157]. These interactions are particularly relevant in patients receiving multi-target neuromodulatory regimens, for example, a patient on β-blockade for LQTS who is prescribed an antidepressant with QT-prolonging potential [158]. Systematic assessment of medication profiles is therefore an integral component of precision neuro-cardiac management.

### Emerging frontiers and future opportunities

#### CNS interventions and gene-based therapies

Central neuromodulatory strategies extend arrhythmia management to higher-order regulatory networks that integrate cardiovascular control and emotional processing. Repetitive transcranial magnetic stimulation (rTMS) and transcranial direct current stimulation (tDCS) are noninvasive brain stimulation techniques that modulate cortical excitability through externally applied magnetic fields or low-intensity electrical currents. Targeted regions commonly include the prefrontal cortex and anterior cingulate cortex, which are functionally linked to emotional regulation and autonomic control. Preliminary investigations suggest that rTMS may attenuate excessive sympathetic drive mediated through the cortex–limbic system–brainstem axis, thereby influencing downstream cardiovascular autonomic output. This conceptual framework raises the possibility that central neuromodulation could serve as an adjunctive strategy in selected patients with refractory paroxysmal AF or symptomatic ventricular ectopy associated with anxiety or depressive disorders. More broadly, these approaches exemplify the emerging strategy of stabilizing cardiac electrophysiology through targeted modulation of central neural circuits [159].

Deep brain stimulation (DBS), widely established in movement disorders such as Parkinson disease, has also attracted interest as a potential modulator of central autonomic networks. By delivering targeted electrical stimulation to limbic and hypothalamic nuclei, DBS may influence higher-order circuits that regulate systemic sympathetic and parasympathetic output. The potential value of DBS for treating extremely refractory sympathetic storm or arrhythmias associated with specific neurocardiogenic syncope remains exploratory [160].

Gene-based modulation of neurotransmitter pathways represents an upstream strategy for reshaping neuro-cardiac interactions. This approach aims to fundamentally regulate the function of the autonomic nervous system or its interaction with the heart by targeting specific genes or molecular pathways. Through vector-mediated delivery systems, the selective overexpression or silencing of genes within the myocardium or associated autonomic ganglia, notably targeting the stellate ganglion, enables the precise manipulation of neurotransmitter synthesis, release, and signaling dynamics. In experimental models of HF, down-regulation of NGF or tyrosine hydroxylase within cardiac tissue or sympathetic nerve terminals has been explored as a means of limiting pathological neural remodeling and aberrant sympathetic sprouting at their source. By attenuating excessive sympathetic innervation, such approaches may mitigate the pro-arrhythmic substrate characteristic of advanced HF and malignant ventricular arrhythmias [160].

Increasing evidence implicates inflammation as a mechanistic bridge linking autonomic dysregulation with arrhythmogenesis. Crosstalk between neural and immune pathways shapes both structural remodeling and electrophysiological instability, thereby contributing to arrhythmic susceptibility. Biological therapies directed against key inflammatory mediators, including IL-1β and TNF-α, have demonstrated cardiovascular effects beyond their primary indications in autoimmune disease. Observational and secondary analyses suggest that attenuation of inflammatory signaling may be accompanied by reductions in arrhythmia burden in selected patient populations [161]. These findings support the concept that pharmacological or biotechnological modulation of the neuro-immune-cardiac axis represents a promising, though still evolving, strategy for arrhythmias associated with inflammatory cardiomyopathy or POAF.

#### Intelligent closed-loop and adaptive bioelectronic systems

Intelligent closed-loop bioelectronic systems, capable of real-time adaptation to neurophysiological states, represent an emerging but largely preclinical frontier. These platforms couple continuous monitoring of autonomic indices (e.g., HRV and baroreflex sensitivity) with algorithms that dynamically adjust stimulation parameters, enabling pre-emptive neuromodulation before overt arrhythmic events. Next-generation multimodal systems integrate electrophysiological monitoring (pacemaker/ICD functions) with autonomic neuromodulation (VNS, SCS), aiming to detect early signatures of sympathetic-vagal imbalance and deliver preventive intervention [162]. While proof-of-concept studies in animal models have been encouraging, these technologies remain untested in clinical populations, and substantial validation is required to determine their therapeutic efficacy and safety.

#### Nanotechnology and high-resolution neural interfacing

Nanotechnology and high-resolution neural interfacing are advancing minimally invasive strategies for cardiac neuromodulation, although all remain at the preclinical stage. Flexible bioelectronic interfaces, including ultrathin, conformable electrodes, can achieve high-spatial-resolution sensing and microstimulation of autonomic fibers, and have shown feasibility in animal models of AF for mapping and selective modulation of intrinsic cardiac plexi [163]. Stimuli-responsive nanomaterials that accumulate near autonomic nerve endings and release neuromodulatory agents upon external (e.g., near-infrared) or endogenous (e.g., ROS) triggers are also under development, offering the conceptual possibility of transient, anatomically confined neural inhibition [164].

Optogenetic tools provide unparalleled spatiotemporal precision for dissecting cardiac neural circuits in preclinical models. Channelrhodopsin-2 (ChR2)-mediated excitation and archaerhodopsin (Arch)-mediated inhibition of stellate ganglia or intrinsic cardiac neurons have been used to demonstrate causal relationships between specific autonomic pathways and arrhythmogenesis [165]. Magnetoelectric nanogenerators (MENGs) have recently enabled wireless, battery-free peripheral nerve stimulation in porcine models [166]. Despite these advances, critical challenges, including long-term biocompatibility, fibrous encapsulation, and the need for precise, stable neural interfaces, must be overcome before clinical translation can be contemplated. Rigorous human safety and regulatory frameworks for these technologies are not yet defined.

Long-term safety remains a primary concern, particularly regarding the risk of chronic neural damage or maladaptive plasticity resulting from sustained autonomic modulation. Beyond these technical and safety constraints, the regulatory landscape for bioelectronic medicine is not yet fully defined; there is a lack of standardized clinical endpoints and safety benchmarks specifically tailored to neural-cardiac interventions [162]. Finally, effective patient selection is essential for clinical adoption. Identifying individuals most likely to respond to specific autonomic interventions, based on their unique physiological and genetic profiles, is necessary to optimize the benefit-to-risk ratio and ensure the success of personalized management methods.

#### Methodological challenges and emerging computational opportunities

The roadmap toward clinical neuromodulation is defined by several unanswered questions and methodological challenges. A primary barrier is the development of robust, long-term interfaces capable of stable sensing and stimulation within the dynamic environment of the heart and nerves. Furthermore, the lack of standardized protocols for neural dosing, including optimal frequency, intensity, and temporal patterns, complicates the comparison of results across different clinical trials.

Future opportunities lie in the integration of artificial intelligence (AI) and machine learning (ML) to refine arrhythmia management. These computational tools are poised to play a transformative role in predicting arrhythmic risk by analyzing high-dimensional datasets from wearable sensors and implanted bioelectronics [167]. ML algorithms can identify subtle autonomic fingerprints, such as specific HRV patterns or neural firing sequences, that precede life-threatening events, enabling prophylactic interventions [168]. Furthermore, AI-driven closed-loop systems could autonomously adjust stimulation parameters in real time based on the physiological state of the individual, effectively transitioning from reactive to predictive neuromodulation. Addressing these methodological hurdles through advanced computation will be essential for the clinical adoption of neural-based therapies.

## Conclusion

Dysregulation of the neuro-cardiac axis, as elaborated throughout this review, redefines cardiac arrhythmias as emergent consequences of network instability driven by spatial heterogeneity, temporal dysregulation, and convergent remodeling. Building on this integrated framework, the therapeutic landscape has expanded from direct rhythm suppression to upstream modulation of the neuro-cardiac interface. Pharmacological, device-based, and behavioral neuromodulatory strategies now offer complementary avenues for restoring autonomic balance and interrupting maladaptive feed-forward loops.

Looking forward, the translation of these mechanistic insights into clinical practice will require overcoming several key challenges. First, the identification and validation of biomarkers capable of capturing real-time neuro-cardiac axis status, such as advanced HRV metrics, neuropeptide signatures, or imaging-based indices of sympathetic innervation, remain essential for patient stratification and treatment monitoring. Second, the development of next-generation closed-loop bioelectronic systems that dynamically adapt stimulation parameters to individual physiological states promises to enhance both efficacy and safety. Third, a deeper understanding of individual neuro-cardiac phenotypes, informed by genetics, autonomic function testing, and neural imaging, will be necessary to guide the selection of mechanism-matched therapies. Ultimately, the vision for precision neuromodulation in arrhythmia management is not merely the suppression of episodic rhythm disturbances, but the sustained restoration of electrophysiological homeostasis through individualized, dynamic recalibration of the neuro-cardiac axis. Achieving this vision will require multidisciplinary collaboration spanning neuroscience, cardiology, bioengineering, and immunology.
